# Associations of plasma biomarkers with longitudinal co-pathologies in Alzheimer’s disease and cerebral small vessel disease comorbidity

**DOI:** 10.1016/j.tjpad.2025.100449

**Published:** 2026-01-01

**Authors:** Jing Yang, Xinyuan Zhao, Yidan Liu, Yangwei Cai, Yuhua Fan

**Affiliations:** a Department of Neurology, The First Affiliated Hospital, Sun Yat-sen University, No.58 Zhongshan Road 2, Guangzhou, 510080, China; b Guangdong Provincial Key Laboratory of Diagnosis and Treatment of Major Neurological Diseases, No.58 Zhongshan Road 2, Guangzhou, 510080, China; c National Key Clinical Department and Key Discipline of Neurology, No.58 Zhongshan Road 2, Guangzhou, 510080, China; d Laboratory of Metabolism and Aging, School of Pharmaceutical Sciences (Shenzhen), Shenzhen Campus of Sun Yat‐sen University, No.66 Gongchang Road, Shenzhen, 518107, China; e Department of Neurology, Chengdu Seventh People’s Hospital, No.1188, Shuangxing Avenue, Chengdu, 610000, China; f Department of Cardiology, Sichuan Academy of Medical Sciences & Sichuan Provincial People's Hospital, No.32, West Section 2, 1st Ring Road, Chengdu, 610000, China

**Keywords:** Alzheimer’s disease, Cerebral small vessel disease, Plasma biomarker, Comorbidity

## Abstract

**Background:**

Plasma glial fibrillary acidic protein (GFAP), neurofilament light (NfL), phosphorylated tau217 (p-tau217), and the β-amyloid (Aβ) 42/40 ratio are emerging indicators of neuroinflammation, neurodegeneration, and AD-specific pathology, while their specific roles within Alzheimer’s disease (AD) and cerebral small vessel disease (CSVD) comorbidity are not fully understood.

**Methods:**

Participants with normal cognition or mild cognitive impairment were drawn from the Alzheimer’s Disease Neuroimaging Initiative database. Multivariable linear regression and linear mixed-effects models were employed to examine associations of baseline plasma biomarkers with neuropathological features and cognition. Furthermore, Cox proportional hazards models assessed the associations of plasma biomarkers with the risk of comorbid AD and CSVD.

**Results:**

In total populations, elevated GFAP and p-tau217 were significantly associated with greater white matter hyperintensity (WMH) burden, hippocampal atrophy, cerebral Aβ burden, and cognitive decline at baseline and with progression over time (|*β*| = 0.007 to 1.670, *p* = 0.047 to <0.0001). Within disease-specific subgroups, GFAP, p-tau217, and Aβ42/40 ratio demonstrated associations with hippocampal atrophy or WMH progression in CSVD (|*β*| = 0.011 to 0.220, *p* = 0.046 to 0.010), whereas GFAP, NfL, p-tau217, and Aβ42/40 ratio were linked to hippocampal atrophy and/or WMH progression in typical AD (|*β*| = 0.013 to 0.191, *p* = 0.044 to 0.0002). For Cox proportional hazards models, p-tau217 demonstrated greater precision in predicting progression to the CSVD phenotype within the AD subgroup (Hazard ratios = 1.267 to 3.811, *p* = 0.046 to 0.034).

**Conclusion:**

These findings underscore the potential role of plasma biomarkers in elucidating the synergistic mechanisms underlying AD and CSVD comorbidity.

## 1 Introduction

Brain health stands as a central focus in healthy aging, with cognitive impairment posing one of the most urgent challenges [1,2]. Alzheimer’s disease (AD) and cerebral small vessel disease (CSVD) serve as the principal contributors to dementia in elderly individuals, [3,4] and frequently coexist clinically [5,6]. Critically, the concomitant presentation of AD and CSVD doubles dementia risk and poses unique therapeutic challenges, necessitating integrated strategies that simultaneously address both pathologies [7,8]. However, current understanding of the precise pathophysiological mechanisms underlying this disease overlap remains incomplete.

The emergence of novel ultra-sensitive blood-based immunoassays for quantifying plasma biomarkers has provided a scalable method to assess heterogeneous pathological processes in AD and CSVD [[9], [10], [11], [12]]. Plasma biomarkers such as glial fibrillary acidic protein (GFAP; astrogliosis), neurofilament light (NfL; neuroaxonal injury), phosphorylated tau217 (p-tau217; tau pathology), and β-amyloid (Aβ) 42/40 ratio (Aβ42/40 ratio; amyloid burden) collectively capture neuroinflammatory, neurodegenerative, and AD-specific pathologies [[13], [14], [15]]. These biomarkers are increasingly acknowledged as critical diagnostic, prognostic, and disease-staging tools for AD [16,17]. Meanwhile, emerging studies have begun to explore their utility in predicting cerebrovascular disease features such as white matter hyperintensity (WMH) progression, [9,18] however, there remains a paucity of research specifically investigating comorbid AD-CSVD. Current evidence inadequately clarifies how plasma biomarker profiles correlate with pathological and cognitive trajectories in comorbid AD-CSVD.

The National Institute on Aging and Alzheimer’s Association (NIA-AA) Research Framework defines AD as a biological continuum initiated by AD neuropathologic change [16]. Within this framework, biomarkers such as cerebrospinal fluid (CSF) total-tau (t-tau)/Aβ42 ratio and amyloid positron emission tomography (PET) provide diagnostic evidence of AD pathophysiology under the AT(N) classification system [16]. In parallel, WMH volume, recognized as the most robust Magnetic Resonance Imaging (MRI) biomarker of vascular cognitive impairment, may exert broader neurobiological effects compared to localized vascular lesions (e.g., lacunes or microbleeds) [19]. Building on these evidences, our study employs AD pathology status (CSF t-tau/Aβ42 ratio or Aβ-PET burden) and WMH volume as classification anchors to stratify participants, with hippocampal volume, CSF t-tau/Aβ42 ratio, and Aβ-PET burden operationalizing AD pathology, while WMH volume reflects CSVD pathology. We systematically evaluated the cross-sectional and longitudinal relationships of plasma biomarkers with both AD/CSVD pathology assessments and cognitive trajectories. Aligned with current therapeutic paradigms focusing on early disease intervention, our cohort prioritized individuals across the preclinical-to-prodromal spectrum, specifically including cognitively normal (CN) and mild cognitive impairment (MCI) populations.

## 2 Methods

### 2.1 Participants

Data were obtained from the Alzheimer’s Disease Neuroimaging Initiative (ADNI) database (http://adni.loni.usc.edu), a public-private partnership established in 2003 under the leadership of Principal Investigator Dr. Michael W. Weiner. ADNI aims to identify clinical, imaging, genetic, and biochemical biomarkers for early detection and monitoring of AD and MCI. Ethical approval for the ADNI study was secured from the institutional review boards at each collaborating site. All participants or their legally authorized surrogates provided written informed consent prior to study participation.

From this database, participants were included if they met the following criteria: (1) baseline Clinical Dementia Rating (CDR) < 1; (2) Hachinski Ischemic Score (HIS) ≤ 4; (3) Geriatric Depression Scale-15 (GDS-15) score <6; (4) availability of baseline brain MRI, CSF biomarker measurements or Aβ-PET scans, neuropsychological assessments, and plasma biomarkers (GFAP, NfL, p-tau217, and Aβ42/40 ratio). The final cohort included 330 participants for cross-sectional analyses (110 with Aβ-PET scans and 298 with CSF biomarker measurement), with subsets completing ≥1 follow-up assessments across modalities: 135 participants underwent brain MRI scans (range 0.25 – 8 years), 86 received lumbar punctures for CSF biomarkers quantification (range 2 – 8.5 years), 31 underwent Aβ-PET examinations (range 2 – 7 years) and 249 completed neuropsychological evaluations (range 0.5 – 11 years) during follow-up. Participant selection process is presented in Figure S1.

Participants were stratified by baseline pathology status: (1) AD pathology defined by CSF t-tau/Aβ42 ratio or Aβ-PET burden, and (2) WMH burden. AD status was defined as AD (CSF t-tau/Aβ42 ratio ≥ 0.26 or cortical/cerebellum standardized uptake value ratio [SUVR] ≥1.17) or non-AD, [20,21] while WMH burden was categorized as high (total intracranial volume-adjusted WMH volume > 0.00321, reflecting CSVD) or low [22]. Based on these dichotomous classifications, four mutually exclusive groups emerged: AD−WMH−: non-AD with low WMH burden; AD−WMH+: non-AD with high WMH burden; AD+WMH−: AD with low WMH burden; AD+WMH+: AD with high WMH burden.

### 2.2 Plasma and CSF biomarkers assessment

All blood samples were collected in EDTA anticoagulant tubes, processed *via* centrifugation to isolate plasma, aliquoted, and stored at −80 °C. On the day of analysis, thawed plasma samples were analyzed using the validated Lumipulse chemiluminescent enzyme immunoassay platform [G1200] for Aβ42, Aβ40, and p-tau217, and the Simoa Quanterix [HD-X] IA platform for NfL and GFAP (protocol details: http://adni.loni.usc.edu). For CSF biomarkers, samples obtained *via* lumbar puncture were then frozen at −80 °C. Thawed CSF aliquots were assayed at the UPenn/ADNI Biomarker Laboratory using the Roche Elecsys β-Amyloid (1–42) CSF kit following manufacturer protocols and published methods, [23,24] with parallel quantification of t-tau concentration.

### 2.3 Apolipoprotein E (APOE) genotype assessment

APOE genotype was evaluated according to the ADNI protocol (http://adni.loni.usc.edu), and participants with at least one APOE *ε*4 allele were classified as APOE *ε*4 carriers.

### 2.4 Neuropsychological testing

Certified raters administered standardized neuropsychological evaluations in accordance with ADNI protocols. For this study, we selected the following assessments for subsequent analyses: the CDR, GDS-15, Mini-Mental State Examination (MMSE), Montreal Cognitive Assessment (MoCA), and domain-specific evaluations of executive function, memory, language, and visuo-spatial function.

### 2.5 Neuroimaging data

All MRI and Aβ-PET examinations were performed according to standardized ADNI imaging protocol. WMH quantification was performed using a Bayesian probabilistic segmentation framework integrating high-resolution 3D T1-weighted and fluid-attenuated inversion recovery (FLAIR) sequences. Native-space T1 images were automatically segmented into grey matter, white matter, and CSF compartments through validated pipelines, with total intracranial volume and hippocampal volume derived from automated segmentation. Subsets of participants underwent 18F-Florbetapir-PET to quantify cortical Aβ deposition. Mean cortical SUVRs were calculated using the cerebellum as the reference region, with target regions encompassing the temporal, anterior cingulate, orbital frontal, posterior cingulate, parietal, and precuneus regions [25]. Comprehensive methodological details of the image processing pipeline, including parameter settings and validation metrics, are documented in http://adni.loni.usc.edu.

### 2.6 Statistical analysis

Neuroimaging and biomarker variables—WMH volume, hippocampal volume, CSF t-tau/Aβ42 ratio, Aβ-PET load, and plasma biomarkers (GFAP, NfL, p-tau217, Aβ42/40 ratio)—were logarithmically transformed due to the skewed distribution, while cognitive scores (MMSE and MoCA) were z transformed for further analyses. Group differences in continuous demographic variables were analyzed using one-way analysis of variance (ANOVA) with Bonferroni post hoc corrections for normally distributed data and Kruskal-Wallis tests with Dunn-Bonferroni adjustments for non-parametric data; categorical variables were compared *via* χ² tests. To assess baseline plasma biomarker differences across groups, multivariable linear regression models adjusted for age, sex, and APOE *ε*4 carrier status were implemented.

Given the heterogeneity in sample sizes between cross-sectional and longitudinal cohorts, we conducted independent analyses for each analytical approach to optimize statistical rigor. Cross-sectional associations between plasma biomarkers (GFAP, NfL, p-tau217, Aβ42/40 ratio) and neuroimaging/CSF measures (WMH volume, hippocampal volume, Aβ-PET burden, and CSF t-tau/Aβ42 ratio) or cognitive performance were evaluated using multivariable linear regression models adjusted for age, sex, and APOE *ε*4 status.

Linear mixed-effects models were implemented to assess the impact of baseline plasma biomarkers on longitudinal trajectories of WMH volume, hippocampal volume, Aβ-PET burden, CSF t-tau/Aβ42 ratio, and cognitive function. Models incorporated participant-specific random intercepts, with fixed effects including age, sex, APOE *ε*4 status, baseline values of the respective outcome variable, and predictor-by-time interaction terms to quantify temporal effect modifications. Parameters were estimated using maximum likelihood. P-values for fixed effects were obtained using Satterthwaite’s approximation for degrees of freedom based on model-based standard errors. Logistic regression modeled comorbidity status (AD+WMH+ vs. AD−WMH−), with receiver-operating characteristic (ROC) curves identifying optimal cutoffs *via* Youden index. Biomarkers were dichotomized at these thresholds for subsequent trajectory analyses of neuroimaging parameters, CSF t-tau/Aβ42 ratio decline, and cognitive deterioration. All models adjusted for baseline covariates and included predictor × time interactions to assess group-dependent progression rates.

To further explore the associations between plasma biomarkers and the risk of AD and CSVD comorbidity, we generated Kaplan-Meier curves comparing cumulative comorbidity incidence across high/low biomarker groups (stratified using optimal cutpoints determined by the survminer package), with between-group differences evaluated *via* log-rank tests. Cox proportional hazards models were used to derive hazard ratios (HRs) with 95 % confidence intervals (CIs) for biomarkers, which were analyzed both as continuous and categorical variables. Adjusted models included age and sex. However, APOE *ε*4 status was not included as a covariate due to insufficient sample size. The proportional hazards assumption was verified using scaled Schoenfeld residuals. To address potential bias due to the limited sample size and low event rates, Firth's penalized likelihood correction was applied to Cox models for GFAP and NfL categorical variables. Partial models were fitted using profile likelihood-based CIs instead of conventional Wald intervals to provide more accurate estimation in small samples.

All statistical analyses were implemented using R software (v4.4.2). Linear regression and mixed-effects models were first applied to the full cohort and then repeated within subgroups. Statistical significance was defined as *p* < 0.05 using two-tailed tests.

## 3 Results

### 3.1 Demographic and clinical characteristics

Baseline characteristics of participants, stratified by AD pathology status and WMH burden, are summarized in Table 1. The AD+WMH+ individuals was significantly older than both the AD−WMH− and AD+WMH− individuals (76.92 ± 6.92 vs 69.09 ± 6.52 and 72.00 ± 7.52 years; *p* < 0.001) and had a higher prevalence of APOE *ε*4 carriers than AD−WMH− and AD−WMH+ groups (61.54 % vs 26.67 % and 14.29 %; *p* < 0.001).

**Table 1:** Participant baseline characteristics.

	AD−WMH− *N=131*	AD−WMH+ *N=39*	AD+WMH− *N=91*	AD+WMH+ *N=69*	*p*	Adjusted *p*
**Demographics**						
Age, years	69.09 (6.52)	75.63 (6.40)^a^	72.00 (7.52)^a^^,^^b^	76.92 (6.92)^a^^,^^c^	<0.001	-
Female ( %)	80 (61.07 %)	15 (38.46 %)	40 (43.96 %)	31 (44.93 %)	0.015	-
Education, years	16.46 (2.35)	16.79 (2.55)	16.58 (2.30)	15.91 (2.53)	0.216	-
*APOE ε*4 carrier ( %)	32 (26.67 %)	5 (14.29 %)	58 (69.88 %)^a^^,^^b^	40 (61.54 %)^a^^,^^b^	<0.001	-
**Vascular risk factors**						
BMI, kg/m2	27.34 (5.43)	28.11 (4.25)	28.08 (18.26)	26.61 (4.91)	0.842	-
Hypertension ( %)	40 (30.53 %)	17 (43.59 %)	30 (32.97 %)	33 (47.83 %)	0.067	-
**CSF biomarkers**						
CSF Aβ42	3.18 (0.17)	3.16 (0.15)	2.81 (0.18)^a^^,^^b^	2.77 (0.17)^a^^,^^b^	<0.001	-
CSF t-tau	2.30 (0.13)	2.31 (0.14)	2.49 (0.17)^a^^,^^b^	2.51 (0.20)^a^^,^^b^	<0.001	-
CSF p-tau	1.24 (0.14)	1.25 (0.16)	1.48 (0.19)^a^^,^^b^	1.50 (0.21)^a^^,^^b^	<0.001	-
T-tau/Aβ42	−0.88 (0.11)	−0.85 (0.11)	−0.32 (0.21)^a^^,^^b^	−0.25 (0.21)^a^^,^^b^	<0.001	-
**Imaging markers**						
WMH	−3.23 (0.57)	−2.21 (0.24)^a^	−3.07 (0.44)^a^^,^^b^	−2.11 (0.30)^a^^,^^c^	<0.001	-
Hippocampus	0.82 (0.06)	0.81 (0.07)	0.79 (0.07)^a^	0.77 (0.07)^a^	<0.001	-
Aβ-PET	0.01 (0.03)	−0.01 (0.04)	0.12 (0.06)^a^^,^^b^	0.13 (0.06)^a^^,^^b^	<0.001	-
**Cognition**						
MMSE z-score	0.43 (0.48)	0.32 (0.70)	−0.28 (1.25)^a^^,^^b^	−0.67 (1.11)^a^^,^^b^^,^^c^	<0.001	-
MoCA z-score	0.45 (0.50)	0.30 (0.78)	−0.21 (1.07)^a^^,^^b^	−0.81 (1.21)^a^^,^^b^^,^^c^	<0.001	-
Executive function	1.17 (0.77)	0.70 (0.84)^a^	0.30 (0.92)^a^	−0.39 (0.94)^a^^,^^b^^,^^c^	<0.001	-
Memory	1.16 (0.79)	0.83 (0.93)	0.21 (1.11)^a^^,^^b^	−0.26 (1.02)^a^^,^^b^^,^^c^	<0.001	-
Language cognition	0.98 (0.78)	0.65 (0.82)	0.30 (0.91)^a^	−0.15 (0.93)^a^^,^^b^^,^^c^	<0.001	-
Visuo-spatial function	0.12 (0.70)	−0.03 (0.84)	−0.22 (0.88)^a^	−0.30 (0.93)^a^	0.002	-
**Plasma biomarkers**						
GFAP	2.03 (0.19)	2.13 (0.19)^a^	2.26 (0.22)^a^^,^^b^	2.35 (0.17)^a^^,^^b^^,^^c^	<0.001	<0.001
NfL	1.17 (0.17)	1.35 (0.18)^a^	1.31 (0.18)^a^	1.41 (0.16)^a^^,^^c^	<0.001	<0.001
P-tau217	−1.02 (0.18)	−0.90 (0.17)^a^	−0.48 (0.34)^a^^,^^b^	−0.34 (0.24)^a^^,^^b^^,^^c^	<0.001	<0.001
Aβ42/40	−1.03 (0.04)	−1.02 (0.05)	−1.10 (0.06)^a^^,^^b^	−1.10 (0.04)^a^^,^^b^	<0.001	<0.001

*Notes*: WMH volume was adjusted for total intracranial volume. CSF Aβ42, CSF t-tau, CSF p-tau, CSF t-tau/Aβ42 ratio, WMH volume, hippocampal volume, Aβ-PET load, GFAP, NfL, p-tau217, and Aβ42/40 ratio were all log transformed; MMSE and MoCA scores were z transformed; Data were presented as mean (SD), n ( %), or median (interquartile range). CSF and plasma biomarkers were measured in pg/mL, brain volume was measured in mL. Differences in plasma biomarkers were adjusted for age, sex, and APOE *ε*4 carrier status.

Abbreviations: Aβ, amyloid beta; AD, Alzheimer’s disease; APOE, apolipoprotein E; BMI: Body Mass Index; CSF, cerebrospinal fluid; GFAP, glial fibrillary acidic protein; PET, positron emission tomography; MMSE, Mini-Mental State Examination; MoCA, Montreal Cognitive Assessment; NfL, neurofilament light; P-tau, phosphorylated tau; SD, standard deviation; T-tau, total tau; WMH, white matter hyperintensities.

a *p*, significantly different from AD−WMH−.

b *p*, significantly different from AD−WMH+.

c *p*, significantly different from AD+WMH−.

Plasma GFAP and p-tau217 levels were significantly higher in the AD+WMH+ group than in the AD−WMH− (both *p* < 0.001), AD−WMH+ (both *p* < 0.001), and AD+WMH− (GFAP: *p* = 0.024; p-tau217: *p* = 0.002) groups (Table 1). Similarly, plasma NfL levels were elevated in the AD+WMH+ group compared to the AD−WMH− (*p* < 0.001) and AD+WMH− groups (*p* = 0.004). Furthermore, the AD+WMH+ group exhibited lower plasma Aβ42/40 ratio relative to both the AD−WMH− (*p* < 0.001) and AD−WMH+ groups (*p* < 0.001).

For the longitudinal cohort, compared with the AD−WMH− group, the AD+WMH+ group exhibited higher baseline levels of GFAP, NfL, p - tau217, and a lower Aβ42/40 ratio. Detailed baseline characteristics of the longitudinal cohort are presented in Tables S1–S8.

### 3.2 Cross-sectional association of plasma biomarkers with neuroimaging outcomes, CSF pathology, and cognitive function

**Fig. 1:**
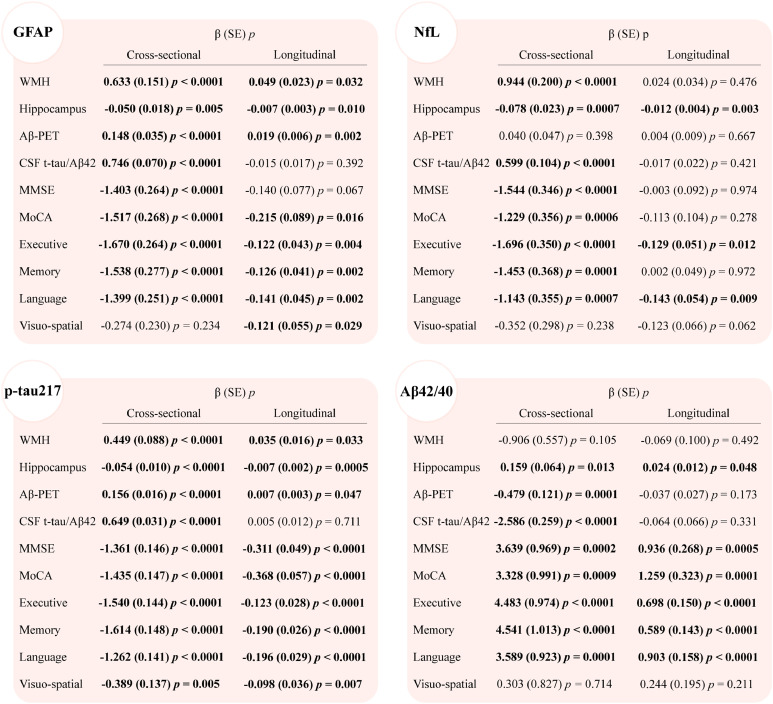
Cross-sectional and longitudinal associations of baseline plasma biomarker with imaging markers, CSF Aβ42/40 ratio, and cognition in the total cohort.

In the full cohort, higher plasma GFAP, NfL, p-tau217, and lower Aβ42/40 ratio demonstrated significant correlations with decreased hippocampal volume, higher CSF t-tau/Aβ42 ratio, and poorer performance in global cognition (MMSE/MoCA) and specific domains including executive function, memory, and language (|*β*| = 0.050 to 4.483, all *p* < 0.05; Fig. 1). Notably, plasma GFAP, NfL, and p-tau217 exhibited positive associations with WMH volumes (*β* = 0.449 to 0.944, all *p* < 0.0001), whereas Aβ42/40 ratio showed no significant association. Higher GFAP, p-tau217, and lower Aβ42/40 ratio were associated with increased Aβ-PET burden (|*β*| = 0.148 to 0.479, all *p* < 0.001), contrasting with NfL's null effect. Plasma p-tau217 specifically showed additional associations with impaired visuo-spatial performance (*β* = −0.389, *p* = 0.005).

WMH volume was adjusted for total intracranial volume. WMH volume, hippocampal volume, GFAP, NfL, p-tau217, and Aβ42/40 ratio were all log transformed; MMSE and MoCA scores were z transformed. Multivariable linear regression models adjusted for age, sex, and APOE ε4 status. Liner mixed models adjusted age, sex, APOE ε4 carrier status, baseline values of the respective outcome variable, and interaction of predictors with time (years). The longitudinal coefficients shown in this figure were for the biomarker × time interaction term. Abbreviations: Aβ, amyloid beta; GFAP, glial fibrillary acidic protein; MMSE, Mini-Mental State Examination; MoCA, Montreal Cognitive Assessment; NfL, neurofilament light; P-tau, phosphorylated tau; T-tau, total tau; WMH, white matter hyperintensities.

The associations between plasma biomarkers and WMH burden, hippocampal volume, and cognition, stratified by AD pathology status, are shown in Table S9. When further stratifying subgroups by AD pathology status and WMH burden revealed distinct patterns: associations between plasma biomarkers and WMH volume were largely non-significant across subgroups (Table S10). The exception was NfL, which remained associated with WMH volume in the AD−WMH− group (*β* = 1.364, *p* < 0.0001). P-tau217 remained uniquely associated with hippocampal volume in both the AD+WMH− (*β* = −0.055, *p* = 0.016) and AD+WMH+ (*β* = −0.095, *p* = 0.005) subgroups. Associations of Aβ-PET burden, CSF t-tau/Aβ42 ratio, and cognitive function with plasma biomarkers across subgroups are detailed in Table S10.

### 3.3 Longitudinal association of plasma biomarkers with neuroimaging outcomes, CSF pathology, and cognitive function

In the full cohort, elevated baseline plasma GFAP and p-tau217 were associated with accelerated WMH progression (GFAP: *β* = 0.049, *p* = 0.032; p-tau217: *β* = 0.035, *p* = 0.033), faster hippocampal atrophy (GFAP: *β* = −0.007, *p* = 0.010; p-tau217: *β* = −0.007, *p* = 0.0005), cerebral Aβ accumulation (GFAP: *β* = 0.019, *p* = 0.002; p-tau217: *β* = 0.007, *p* = 0.047), and cognitive decline across MoCA, executive function, memory, language and visuo-spatial domains (*β* = −0.098 to −0.368, all *p* < 0.05; Fig. 1). Concurrently, higher plasma NfL (*β* = −0.012, *p* = 0.003) and lower Aβ42/40 ratio (*β* = 0.024, *p* = 0.048) independently predicted faster hippocampal atrophy. Elevated NfL levels correlated with longitudinal decline in executive function (*β* = −0.129, *p* = 0.012) and language (*β* = −0.143, *p* = 0.009), whereas reduced Aβ42/40 ratio showed broader associations, encompassing deterioration in MMSE (*β* = 0.936, *p* = 0.0005), MoCA (*β* = 1.259, *p* = 0.0001), executive function (*β* = 0.698, *p* < 0.0001), memory (*β* = 0.589, *p* < 0.0001), and language performance (*β* = 0.903, *p* < 0.0001).

An exploration of the associations between baseline plasma biomarkers and longitudinal WMH burden, hippocampal volume, and cognition, stratified by AD pathology status, is provided in Table S11. Subsequent subgroup analyses revealed distinct pathological trajectories. Within the AD−WMH+ group, p-tau217 was associated with WMH progression (*β* = 0.220, *p* = 0.010), while GFAP (*β* = −0.011, *p* = 0.046) and Aβ42/40 ratio (*β* = 0.069, *p* = 0.043) were associated with hippocampal atrophy (Table 2). Among AD+WMH− participants, elevated GFAP, NfL, and p-tau217 were associated with both WMH progression and hippocampal atrophy (|*β*| = 0.013 to 0.191, all *p* < 0.05), and a reduced Aβ42/40 ratio was associated with hippocampal atrophy (*β* = 0.056, *p* = 0.044). Conversely, no significant associations between plasma biomarkers and WMH progression or hippocampal atrophy were observed in the AD−WMH− or AD+WMH+ groups. Associations between plasma biomarkers and longitudinal cognitive function were also explored (Table S12).

**Table 2:** Longitudinal associations of baseline plasma biomarkers with imaging markers in subgroups.

	GFAP*time	NfL*time	p-tau217*time	Aβ42/40*time
	β (SE) *p*	β (SE) *p*	β (SE) *p*	β (SE) *p*
**WMH**				
AD−WMH−	0.008 (0.060) 0.897	−0.093 (0.078) 0.235	0.027 (0.072) 0.711	0.048 (0.272) 0.861
AD−WMH+	0.058 (0.059) 0.330	0.094 (0.075) 0.214	0.220 (0.082) 0.010	−0.601 (0.337) 0.081
AD+WMH−	0.098 (0.041) 0.018	0.191 (0.054) 0.0007	0.059 (0.026) 0.026	−0.158 (0.193) 0.415
AD+WMH+	−0.024 (0.047) 0.608	−0.029 (0.051) 0.569	0.017 (0.021) 0.414	−0.138 (0.188) 0.466
**Hippocampus**				
AD−WMH−	0.007 (0.005) 0.138	−0.008 (0.006) 0.220	0.0002 (0.006) 0.973	−0.043 (0.023) 0.058
AD−WMH+	−0.011 (0.005) 0.046	−0.006 (0.007) 0.416	0.005 (0.009) 0.592	0.069 (0.033) 0.043
AD+WMH−	−0.022 (0.006) 0.0002	−0.019 (0.008) 0.025	−0.013 (0.004) 0.0003	0.056 (0.027) 0.044
AD+WMH+	−0.012 (0.008) 0.164	−0.014 (0.009) 0.118	−0.0004 (0.004) 0.915	−0.027 (0.034) 0.429

*Notes*: WMH volume was adjusted for total intracranial volume. WMH volume, hippocampal volume, GFAP, NfL, p-tau217, and Aβ42/40 ratio were all log transformed; Liner mixed models adjusted age, sex, APOE *ε*4 carrier status, baseline values of the respective outcome variable, and interaction of predictors with time (years).

Abbreviations: Aβ, amyloid beta; AD, Alzheimer’s disease; GFAP, glial fibrillary acidic protein; NfL, neurofilament light; P-tau, phosphorylated tau; T-tau, total tau; WMH, white matter hyperintensities.

Youden's index-optimized cutoffs (derived from AD−WMH− vs. AD+WMH+ group comparisons) were employed to stratify baseline plasma biomarkers (Table S13, Figure S2), then systematically evaluated their longitudinal associations with neuroimaging trajectories, CSF t-tau/Aβ42 ratio dynamics, and multidomain cognitive changes in the total cohort, as visualized in Figure S3.

### 3.4 Longitudinal associations of plasma biomarkers with incident comorbid AD-CSVD

**Fig. 2:**
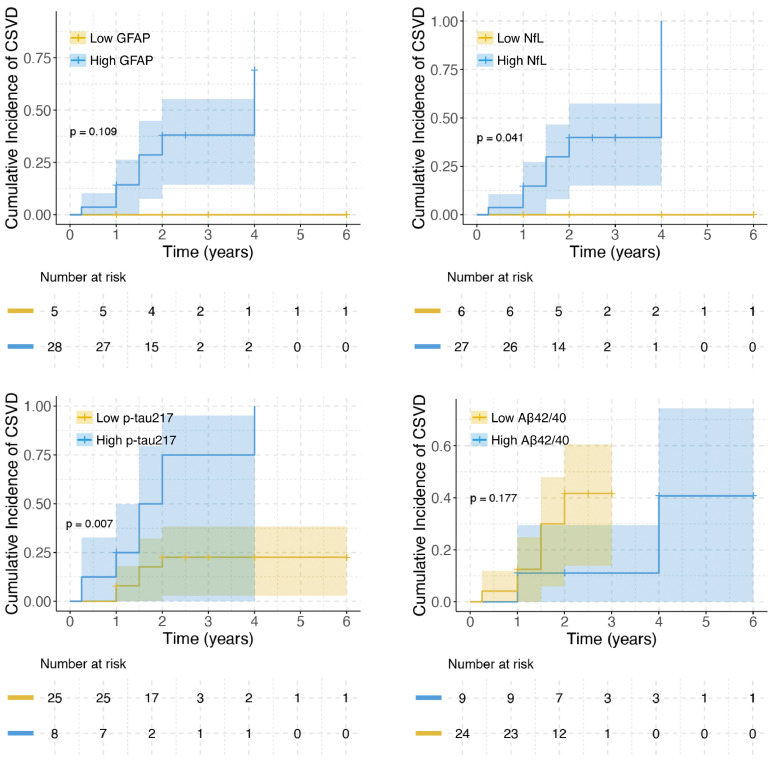
Kaplan–Meier curve of comorbid AD-CSVD incidence in AD.

Among 33 AD+WMH− participants, 10 progressed to CSVD (WMH+) over 6 years. Kaplan-Meier curves demonstrated significantly different time-to-comorbidity for p-tau217 (cut point = 0.42 pg/mL; *p* = 0.007) and NfL (cut point = 15.5 pg/mL; *p* = 0.041), while stratification by GFAP (cut point = 117.5 pg/mL) and Aβ42/40 ratio (cut point = 0.08) showed non-significant separation (Fig. 2). Cox regression revealed each 0.1-unit increase in continuous p-tau217 conferred 27 % higher risk in unadjusted models (HR = 1.267, *p* = 0.034) and 29 % higher risk in age/sex-adjusted models (HR = 1.289, *p* = 0.036; Table 3). Categorically, high p-tau217 showed ∼3.8-fold increased risk in both univariable (HR = 3.738, *p* = 0.039) and multivariable analyses (HR = 3.811, *p* = 0.046). High NfL was associated with increased risk categorically (univariable HR = 9.866, *p* = 0.038; multivariable HR = 14.163, *p* = 0.019). Neither GFAP nor Aβ42/40 ratio showed significant associations.

Biomarker cutpoints were identified using the survminer package in R. Group differences were evaluated *via* log-rank tests. Aβ, amyloid beta; GFAP, glial fibrillary acidic protein; NfL, neurofilament light; P-tau, phosphorylated tau.

**TABLE 3:** Hazard Ratios (95 %CI) for comorbid AD and CSVD in AD.

	Continuous Variable			Categorical Variable*	
	HR (95 %CI)	*p*		HR (95 %CI)	*p*
**Univariable model**					
GFAP, per unit	1.002 (0.997–1.008)	0.426	GFAP†	5.134 (1.917–8.213e+287)‡	0.146
NfL, per unit	1.006 (0.941–1.076)	0.865	NfL†	9.866 (3.004-Inf)‡	0.038
p-tau217, per 0.1 unit	1.267 (1.018–1.577)	0.034	p-tau217	3.738 (1.063–13.150)	0.039
Aβ42/40, per 0.01 unit	0.842 (0.384–1.846)	0.668	Aβ42/40	3.774 (0.470–30.293)	0.211
**Multivariable model**					
GFAP, per unit	1.002 (0.995–1.009)	0.514	GFAP†	4.679 (1.308–40.774)‡	0.187
NfL, per unit	1.001 (0.929–1.078)	0.976	NfL†	14.163 (4.171-Inf)‡	0.019
p-tau217, per 0.1 unit	1.289 (1.016–1.635)	0.036	p-tau217	3.811 (1.026–14.152)	0.046
Aβ42/40, per 0.01 unit	0.837 (0.380–1.842)	0.658	Aβ42/40	3.962 (0.489–32.097)	0.197

*Notes*: Multivariable models adjusted age and sex. Plasma biomarkers were measured in pg/mL. Biomarker cutpoints were identified using the survminer package in R.

Abbreviations: Aβ, amyloid beta; CI, Confidence interval; GFAP, glial fibrillary acidic protein; HR, Hazard ratio; NfL, neurofilament light; P-tau, phosphorylated tau; T-tau, total tau.

⁎ Stratified using optimal cutpoints determined by the survminer package.

† Cox regression models were fitted using Firth's bias-reduced penalized likelihood method.

‡ Profile likelihood CI.

Survival analysis was not performed for 12 AD−WMH+ participants with longitudinal CSF t-tau/Aβ42 or Aβ-PET due to limited sample size. Among normal controls, survival analysis including Kaplan-Meier curves and Cox regression was conducted for 46 participants with longitudinal WMH volume data (Figure S4, Table S14). For 44 normal controls with longitudinal CSF t-tau/Aβ42 or Aβ-PET, survival analysis was not performed given only 3 AD conversions.

## 4 Discussion

This study investigated cross-sectional and longitudinal associations between plasma biomarkers (GFAP, NfL, p-tau217, Aβ42/40 ratio), co-pathology progression (WMH volume, hippocampal atrophy, Aβ-PET burden, CSF t-tau/Aβ42 ratio), and cognitive trajectories in individuals with CN and MCI. Key findings revealed: (1) In the total population, GFAP and p-tau217 levels were significantly associated with the progression of co-pathologies (WMH volume, hippocampal volume, Aβ-PET burden) and cognitive decline trajectories; (2) In the typical AD subgroup (AD+WMH−), elevated GFAP, NfL, and p-tau217 levels were associated with WMH progression, with p-tau217 demonstrating higher precision in predicting longitudinal conversion to the CSVD phenotype; (3) In the CSVD subgroup (AD−WMH+), elevated GFAP and reduced Aβ42/40 ratio were significantly associated with hippocampal atrophy. These findings underscore the potential role of plasma biomarkers in elucidating the synergistic mechanisms underlying AD and CSVD comorbidity.

Emerging evidences suggest that plasma GFAP and NfL are associated with WMH progression [9,10,26,27] and AD pathology, [10,28] while p-tau217 and Aβ42/40 ratio have been proposed as AD-specific biomarkers [29] with recent studies indicating their dual roles in both AD [15,30] and CSVD pathology [9]. In this study, baseline comparisons revealed significantly elevated plasma GFAP and p-tau217 in AD+WMH+ individuals versus other subgroups, preliminarily suggesting their association with AD and CSVD comorbidity. In the total populations, cross-sectional and longitudinal analyses demonstrated robust correlations of GFAP and p-tau217 with WMH progression, hippocampal atrophy, and global cognitive decline. While our findings align with some previous reports, [9,[31], [32], [33], [34], [35]] contrasting studies showing no association between GFAP and hippocampal volume [35,36]. This discrepancy may arise from either their participants being in the very early stage of AD pathology or shorter follow-up durations limiting detection of delayed biomarker-pathology associations. Elevated GFAP levels, which are thought to represent reactive astrogliosis and commonly used as a biomarker of reactive neuroinflammation, [37,38] may exacerbate neuronal injury and synaptic loss, [39] thereby accelerating hippocampal atrophy. Notably, p-tau217 exhibited an association with WMH volume, which has been specifically linked to the cerebral amyloid angiopathy (CAA), [40,41] suggesting its potential role in aggravating WMH through CAA-related vascular destruction [42,43]. In this study, plasma GFAP and p-tau217 showed robust associations with concurrent cerebral Aβ burden and CSF t-tau/Aβ42 ratio at baseline, while these associations were not sustained longitudinally with CSF t-tau/Aβ42 progression. Previous study has demonstrated their longitudinal correlations with cerebral Aβ accumulation dynamics [28,44,45]. Although the NIA-AA criteria propose that both Aβ-PET and the CSF t-tau/Aβ42 ratio can be utilized for diagnosing AD, it is important to note that PET and CSF-based measurements are not equivalent [16]. Our preliminary analyses in the total cohort indicated that GFAP and p-tau217 are associated with both the initiation and dynamic progression of AD-CSVD co-pathologies.

Existing studies predominantly analyze plasma biomarkers as continuous variables to detect linear associations, [46,47] with limited exploration of threshold-based biomarker-pathology relationships, as few studies stratify biomarkers into high/low groups using predefined clinical thresholds [28]. In the total population, continuous plasma NfL was associated with WMH volume cross-sectionally, but this association was absent in longitudinal analyses, while prior studies report associations between continuous NfL and WMH progression [9,10]. When dichotomizing baseline NfL into high/low groups *via* Youden’s index-derived thresholds, we found that higher NfL predicted WMH progression. This threshold-dependent significance suggests that NfL predominantly impacts WMH pathology when exceeding a critical concentration. Notably, our findings align with existing literature demonstrating NfL's association with hippocampal atrophy and cognitive decline [10] .

For the Aβ42/40 ratio, significant cross-sectional and longitudinal associations with hippocampal atrophy and cognitive decline were observed in the total population, while no association emerged with WMH progression. Some publications suggest Aβ pathology may contribute to WMH development, [9,48] but the Aβ-vascular pathology relationship remains controversial [49,50]. An alternative explanation posits that WMH primarily stems from uncontrolled vascular risk factors and operates independently of Aβ pathology [51] .

Stratified analyses further clarified the expression patterns of co-pathologies, with specific key correlation differences among subgroups detailed as follows:

First, in the AD participants, elevated baseline plasma GFAP, NfL, and p-tau217 were significantly associated with the progression of WMH. Further stratification based on baseline WMH burden revealed that these associations were exclusively observed in the typical AD subgroup (AD+WMH−). Notably, Cox regression analysis identified p-tau217 as the most precise predictor of longitudinal conversion to AD and CSVD comorbidity. Collectively, these findings underscore the pivotal roles of GFAP, NfL, and particularly p-tau217 in the progression from typical AD to comorbid AD-CSVD. In addition, the Aβ42/40 ratio—along with several other biomarkers—may also reflect ongoing AD-related neurodegenerative processes.

Second, in the CSVD subgroup (AD−WMH+), elevated baseline plasma GFAP and reduced Aβ42/40 ratio were associated with hippocampal atrophy, suggesting a potential role for these biomarkers in the transition from isolated CSVD to comorbid AD-CSVD.

Finally, in the comorbid AD-CSVD (AD+WMH+), there were no associations between all plasma biomarkers with WMH progression and hippocampal atrophy. A potential explanation for these findings is that cerebrovascular injury in CSVD may accelerate Aβ deposition and tau phosphorylation in AD, [52] while AD-related neurodegeneration conversely exacerbates vascular dysfunction [53]. This bidirectional interaction may induce nonlinear biomarker dynamics that obscure pathology-specific correlations typically observed in isolated diseases and necessitating future verification. Additionally, CSVD-driven vascular inflammation and AD-associated neuroinflammation may mutually amplify astrocytic and microglial hyperactivity, [54,55] leading to a "ceiling effect" or dilution of biomarker-structural injury correlations by confounding inflammatory signals. Furthermore, considering that Aβ accumulation begins very early in the AD continuum, [56] with substantial plasma biomarker changes often occurring preclinically, [57] the comorbid group might represent a later stage where biomarker levels have plateaued or exhibit more complex dynamics, reducing their correlation with ongoing structural changes.

Therefore, plasma biomarkers can play an important role by strategically complementing existing methods, primarily by improving detection accessibility and enabling a more dynamic assessment of co-pathology progression. PET and CSF testing are costly, have limited accessibility, and are invasive, which poses significant barriers to their widespread and repeated use. In this context, plasma testing offers a scalable and cost-effective alternative for initial screening, risk stratification, and longitudinal monitoring.

However, some limitations should be acknowledged in our study. First, our focus on WMH as the representative CSVD marker did not incorporate other pathological features (e.g., microbleeds or infarcts), potentially oversimplifying CSVD complexity. Second, the retrospective design using ADNI data may introduce selection biases. Furthermore, the restricted sample size limits statistical power to detect subtle associations. Although this study has uncovered important longitudinal biomarker-pathology associations, because of its observational nature, we cannot fully establish causality. Therefore, future validation studies and additional omics investigations are essential to confirm and expand our understanding of mechanisms underlying AD and CSVD co-pathologies.

In conclusion, this study clarifies how plasma biomarkers track the progression of AD and CSVD co-pathology over time, demonstrating their potential as complementary clinical tools for prognostic prediction and monitoring pathological and cognitive progression in AD and CSVD comorbidity.

## 5 Study funding

Study design and data analysis for this project was supported by grants from the National Natural Science Foundation of China (No.82471306); Noncommunicable Chronic Diseases-National Science and Technology Major Project (2023ZD0504800, 2023ZD0504804); Guangdong Provincial Clinical Research Center for Neurological Diseases (2020B1111170002); Guangdong Province International Cooperation Base for Early Intervention and Functional Rehabilitation of Neurological Diseases (2020A0505020004); Guangzhou Major Difficult and Rare Diseases Project (2024MDRD02); Guangdong Provincial Engineering Center for Major Neurological Disease Treatment; Guangdong Provincial Translational Medicine Innovation Platform for Diagnosis and Treatment of Major Neurological Disease; Guangzhou Clinical Research and Translational Center for Major Neurological Diseases.

## CRediT authorship contribution statement

**Jing Yang:** Writing – original draft, Visualization, Validation, Software, Methodology, Formal analysis, Data curation, Conceptualization. **Xinyuan Zhao:** Writing – original draft, Visualization, Validation, Methodology, Formal analysis, Data curation. **Yidan Liu:** Writing – review & editing, Validation, Methodology, Formal analysis. **Yangwei Cai:** Writing – review & editing, Software, Formal analysis. **Yuhua Fan:** Writing – review & editing, Supervision, Resources, Project administration, Funding acquisition, Conceptualization.

## Declaration of competing interest

The authors declare that they have no known competing financial interests or personal relationships that could have appeared to influence the work reported in this paper.

## Appendix Supplementary materials

Image, application 1

Image, application 2
