# Supplementary material for: Associations of plasma biomarkers with longitudinal co-pathologies in Alzheimer’s disease and cerebral small vessel disease comorbidity
Source: J Prev Alzheimers Dis. 2026 Jan 1;13(2):100449. doi: 10.1016/j.tjpad.2025.100449 (PMC12869035; doi:10.1016/j.tjpad.2025.100449)
Supplement: Supplementary file 1 [file mmc1.docx]

**TABLE S1** Baseline characteristics of the longitudinal cohort: Association between baseline plasma biomarkers and MRI structural changes.

|  | AD−WMH−  *N=46* | AD−WMH+  *N=21* | AD+WMH−  *N=33* | AD+WMH+  *N=35* | *p* | Adjusted *p* |
| --- | --- | --- | --- | --- | --- | --- |
| **Demographics** |  |  |  |  |  |  |
| Age, years | 69.54 (6.99) | 76.34 (5.31)^a^ | 73.22 (7.20) | 78.11 (6.36)^a,c^ | <0.001 | - |
| Female (%) | 27 (58.70%) | 8 (38.10%) | 18 (54.55%) | 15 (42.86%) | 0.313 | - |
| Education, years | 16.13 (2.41) | 16.86 (2.87) | 17.00 (2.28) | 15.94 (2.14) | 0.199 | - |
| *APOE ε*4 carrier (%) | 12 (26.09%) | 2 (10.00%) | 22 (68.75%)^a,b^ | 18 (51.43%)^b^ | <0.001 | - |
| **CSF biomarkers** |  |  |  |  |  |  |
| CSF Aβ42 | 3.16 (0.18) | 3.21 (0.15) | 2.83 (0.17)^a,b^ | 2.81 (0.14)^a,b^ | <0.001 | - |
| CSF t-tau | 2.28 (0.13) | 2.36 (0.13) | 2.49 (0.14)^a,b^ | 2.52 (0.17)^a,b^ | <0.001 | - |
| CSF p-tau | 1.21 (0.15) | 1.29 (0.15) | 1.47 (0.16)^a,b^ | 1.51 (0.19)^a,b^ | <0.001 | - |
| T-tau/Aβ42 | -0.88 (0.11) | -0.86 (0.11) | -0.34 (0.21)^a,b^ | -0.29 (0.20)^a,b^ | <0.001 | - |
| **Imaging markers** |  |  |  |  |  |  |
| WMH | -3.20 (0.50) | -2.27 (0.25)^a^ | -3.01 (0.40)^b^ | -2.13 (0.29)^a,c^ | <0.001 | - |
| Hippocampus | 0.82 (0.06) | 0.82 (0.07) | 0.79 (0.08) | 0.77 (0.06)^a^ | 0.005 | - |
| Aβ-PET | -0.01 (0.03) | -0.01 (0.04) | 0.11 (0.07)^a,b^ | 0.12 (0.07)^a,b^ | <0.001 | - |
| **Cognition** |  |  |  |  |  |  |
| MMSE z-score | 0.39 (0.47) | 0.38 (0.54) | -0.14 (1.31) | -0.60 (1.09)^a,b^ | <0.001 | - |
| MoCA z-score | 0.39 (0.52) | 0.26 (0.77) | -0.02 (1.06) | -0.68 (1.22)^a,b,c^ | <0.001 | - |
| Executive function | 1.21 (0.78) | 0.92 (0.76) | 0.48 (0.94)^a^ | -0.42 (0.91)^a,b,c^ | <0.001 | - |
| Memory | 1.28 (0.79) | 0.89 (0.91) | 0.46 (1.07)^a^ | -0.19 (1.15)^a,b,c^ | <0.001 | - |
| Language cognition | 0.94 (0.86) | 0.63 (0.78) | 0.66 (0.97) | -0.24 (1.00)^a,b,c^ | <0.001 | - |
| Visuo-spatial function | 0.01 (0.76) | 0.10 (0.72) | -0.11 (0.93) | -0.24 (0.88) | 0.416 | - |
| **Plasma biomarkers** |  |  |  |  |  |  |
| GFAP | 2.00 (0.19) | 2.14 (0.20)^a^ | 2.23 (0.22)^a^ | 2.33 (0.18)^a,b^ | <0.001 | <0.001 |
| NfL | 1.17 (0.17) | 1.34 (0.16)^a^ | 1.32 (0.17)^a^ | 1.40 (0.15)^a^ | <0.001 | 0.001 |
| P-tau217 | -1.05 (0.16) | -0.91 (0.15) | -0.57 (0.30)^a,b^ | -0.39 (0.28)^a,b,c^ | <0.001 | <0.001 |
| Aβ42/40 | -1.03 (0.05) | -1.01 (0.04) | -1.10 (0.06)^a,b^ | -1.11 (0.04)^a,b^ | <0.001 | <0.001 |

*Notes*: WMH volume was adjusted for total intracranial volume. CSFAβ42, CSF t-tau, CSF p-tau, CSF t-tau/Aβ42 ratio, WMH volume, hippocampal volume, Aβ-PET load, GFAP, NfL, p-tau217, and Aβ42/40 ratio were all log transformed; MMSE and MoCA scores were z transformed; Data were presented as mean (SD), n (%), or median (interquartile range). CSF and plasma biomarkers were measured in pg/mL, brain volume was measured in mL. Differences in plasma biomarkers were adjusted for age, sex, and APOE *ε*4 carrier status.

Abbreviations: Aβ, amyloid beta; AD, Alzheimer’s disease; APOE, apolipoprotein E; CSF, cerebrospinal fluid; GFAP, glial fibrillary acidic protein; PET, positron emission tomography; MMSE, Mini-Mental State Examination; MoCA, Montreal Cognitive Assessment; NfL, neurofilament light; P-tau, phosphorylated tau; SD, standard deviation; T-tau, total tau; WMH, white matter hyperintensities.

^a^*p*, significantly different from AD−WMH−.

^b^*p*, significantly different from AD−WMH+.

^c^*p*, significantly different from AD+WMH−.

**TABLE S2** Participant MRI follow-up

|  | N = 135 |
| --- | --- |
| No. of longitudinal MRI assessment | 1.80 (0.80; 1 to 5) |
| Duration of MRI follow-up, years | 1.90 (1.40; 0.25 to 8.00) |

*Notes*: Data were presented as mean (SD; range)

**TABLE S3** Baseline characteristics of the longitudinal cohort: Association between baseline plasma biomarkers and cognition changes.

|  | AD−WMH−  *N=98* | AD−WMH+  *N=31* | AD+WMH−  *N=67* | AD+WMH+  *N=53* | *p* | Adjusted *p* |
| --- | --- | --- | --- | --- | --- | --- |
| **Demographics** |  |  |  |  |  |  |
| Age, years | 69.28 (6.54) | 75.22 (6.18)^a^ | 71.84 (7.19) | 77.77 (6.90)^a,c^ | <0.001 | - |
| Female (%) | 58 (59.18%) | 12 (38.71%) | 32 (47.76%) | 22 (41.51%) | 0.088 | - |
| Education, years | 16.39 (2.35) | 17.00 (2.57) | 16.70 (2.42) | 15.87 (2.57) | 0.151 | - |
| *APOE ε*4 carrier (%) | 26 (27.96%) | 3 (10.71%) | 42 (68.85%)^a,b^ | 27 (54.00%)^a,b^ | <0.001 | - |
| **CSF biomarkers** |  |  |  |  |  |  |
| CSF Aβ42 | 3.18 (0.16) | 3.20 (0.14) | 2.81 (0.17)^a,b^ | 2.77 (0.15)^a,b^ | <0.001 | - |
| CSF t-tau | 2.30 (0.13) | 2.32 (0.14) | 2.47 (0.17)^a,b^ | 2.51 (0.19)^a,b^ | <0.001 | - |
| CSF p-tau | 1.24 (0.15) | 1.25 (0.16) | 1.46 (0.19)^a,b^ | 1.51 (0.21)^a,b^ | <0.001 | - |
| T-tau/Aβ42 | -0.88 (0.11) | -0.88 (0.10) | -0.34 (0.22)^a,b^ | -0.26 (0.20)^a,b^ | <0.001 | - |
| **Imaging markers** |  |  |  |  |  |  |
| WMH | -3.22 (0.56) | -2.25 (0.26)^a^ | -3.08 (0.47)^b^ | -2.15 (0.28)^a,c^ | <0.001 | - |
| Hippocampus | 0.83 (0.05) | 0.81 (0.07) | 0.79 (0.07)^a^ | 0.78 (0.07)^a^ | <0.001 | - |
| Aβ-PET | <0.01 (0.03) | -0.01 (0.04) | 0.11 (0.06)^a,b^ | 0.13 (0.06)^a,b^ | <0.001 | - |
| **Cognition** |  |  |  |  |  |  |
| MMSE z-score | 0.40 (0.49) | 0.34 (0.74) | -0.19 (1.21)^a,b^ | -0.69 (1.11)^a,b,c^ | <0.001 | - |
| MoCA z-score | -0.05 (0.80) | -0.34 (1.27) | -0.93 (1.58)^a^ | -1.96 (1.96)^a,b,c^ | <0.001 | - |
| Executive function | 1.14 (0.76) | 0.88 (0.75) | 0.36 (0.88)^a,b^ | -0.39 (0.90)^a,b,c^ | <0.001 | - |
| Memory | 1.18 (0.78) | 0.89 (0.92) | 0.37 (1.05)^a,b^ | -0.21 (1.00)^a,b,c^ | <0.001 | - |
| Language cognition | 0.96 (0.81) | 0.74 (0.84) | 0.40 (0.94)^a^ | -0.16 (0.96)^a,b,c^ | <0.001 | - |
| Visuo-spatial function | 0.13 (0.71) | -0.01 (0.80) | -0.11 (0.87) | -0.23 (0.89)^a^ | 0.047 | - |
| **Plasma biomarkers** |  |  |  |  |  |  |
| GFAP | 2.04 (0.20) | 2.14 (0.19) | 2.24 (0.22)^a^ | 2.34 (0.19)^a,b,c^ | <0.001 | <0.001 |
| NfL | 1.16 (0.17) | 1.33 (0.16)^a^ | 1.32 (0.19)^a^ | 1.40 (0.15)^a^ | <0.001 | <0.001 |
| P-tau217 | -1.02 (0.18) | -0.92 (0.17) | -0.53 (0.35)^a,b^ | -0.38 (0.26)^a,b,c^ | <0.001 | <0.001 |
| Aβ42/40 | -1.03 (0.04) | -1.01 (0.04) | -1.11 (0.05)^a,b^ | -1.10 (0.04)^a,b^ | <0.001 | <0.001 |

*Notes*: WMH volume was adjusted for total intracranial volume. CSFAβ42, CSF t-tau, CSF p-tau, CSF t-tau/Aβ42 ratio, WMH volume, hippocampal volume, Aβ-PET load, GFAP, NfL, p-tau217, and Aβ42/40 ratio were all log transformed; MMSE and MoCA scores were z transformed; Data were presented as mean (SD), n (%), or median (interquartile range). CSF and plasma biomarkers were measured in pg/mL, brain volume was measured in mL. Differences in plasma biomarkers were adjusted for age, sex, and APOE *ε*4 carrier status.

Abbreviations: Aβ, amyloid beta; AD, Alzheimer’s disease; APOE, apolipoprotein E; CSF, cerebrospinal fluid; GFAP, glial fibrillary acidic protein; PET, positron emission tomography; MMSE, Mini-Mental State Examination; MoCA, Montreal Cognitive Assessment; NfL, neurofilament light; P-tau, phosphorylated tau; SD, standard deviation; T-tau, total tau; WMH, white matter hyperintensities.

^a^*p*, significantly different from AD−WMH−.

^b^*p*, significantly different from AD−WMH+.

^c^*p*, significantly different from AD+WMH−.

**TABLE S4** Participant cognition follow-up

|  | N = 249 |
| --- | --- |
| No. of longitudinal cognitive assessment | 2.10 (1.45; 1 to 10) |
| Duration of cognition follow-up, years | 2.47 (1.80; 0.5 to 11) |

*Notes*: Data were presented as mean (SD; range)

**TABLE S5** Baseline characteristics of the longitudinal cohort: Association between baseline plasma biomarkers and Aβ-PET changes.

|  | TOTAL  *N=31* |
| --- | --- |
| **Demographics** |  |
| Age, years | 76.34 (6.43) |
| Female (%) | 10 (32.26%) |
| Education, years | 16.48 (3.08) |
| *APOE ε*4 carrier (%) | 14 (45.16%) |
| **CSF biomarkers** |  |
| CSF Aβ42 | 2.99 (0.25) |
| CSF t-tau | 2.44 (0.21) |
| CSF p-tau | 1.41 (0.24) |
| T-tau/Aβ42 | -0.55 (0.34) |
| **Imaging markers** |  |
| WMH | -2.62 (0.55) |
| Hippocampus | 0.82 (0.04) |
| Aβ-PET | 0.07 (0.09) |
| **Cognition** |  |
| MMSE | 28.10 (1.92) |
| MoCA | 25.81 (3.13) |
| Executive function | 0.39 (0.90) |
| Memory | 0.54 (0.88) |
| Language cognition | 0.51 (0.78) |
| Visuo-spatial function | -0.17 (0.73) |
| **Plasma biomarkers** |  |
| GFAP | 2.16 (0.21) |
| NfL | 1.28 (0.14) |
| P-tau217 | -0.69 (0.31) |
| Aβ42/40 | -1.07 (0.05) |

*Notes*: WMH volume was adjusted for total intracranial volume. CSFAβ42, CSF t-tau, CSF p-tau, CSF t-tau/Aβ42 ratio, WMH volume, hippocampal volume, Aβ-PET load, GFAP, NfL, p-tau217, and Aβ42/40 ratio were all log transformed; MMSE and MoCA scores were z transformed; Data were presented as mean (SD), n (%), or median (interquartile range). CSF and plasma biomarkers were measured in pg/mL, brain volume was measured in mL.

Abbreviations: Aβ, amyloid beta; AD, Alzheimer’s disease; APOE, apolipoprotein E; CSF, cerebrospinal fluid; GFAP, glial fibrillary acidic protein; PET, positron emission tomography; MMSE, Mini-Mental State Examination; MoCA, Montreal Cognitive Assessment; NfL, neurofilament light; P-tau, phosphorylated tau; SD, standard deviation; T-tau, total tau; WMH, white matter hyperintensities.

**TABLE S6** Participant Aβ-PET follow-up

|  | N = 31 |
| --- | --- |
| No. of longitudinal Aβ-PET assessment | 1.39 (0.67; 1 to 3) |
| Duration of Aβ-PET follow-up, years | 2.86 (1.42; 2 to 7) |

*Notes*: Data were presented as mean (SD; range)

**TABLE S7** Baseline characteristics of the longitudinal cohort: Association between baseline plasma biomarkers and CSF t-tau/Aβ42 changes.

|  | TOTAL  *N=86* |
| --- | --- |
| **Demographics** |  |
| Age, years | 72.29 (6.20) |
| Female (%) | 47 (54.65%) |
| Education, years | 16.65 (2.42) |
| *APOE ε*4 carrier (%) | 14 (45.16%) |
| **CSF biomarkers** |  |
| CSF Aβ42 | 3.01 (0.22) |
| CSF t-tau | 2.37 (0.19) |
| CSF p-tau | 1.33 (0.23) |
| T-tau/Aβ42 | -0.63 (0.33) |
| **Imaging markers** |  |
| WMH | -2.84 (0.56) |
| Hippocampus | 0.82 (0.06) |
| Aβ-PET | 0.07 (0.08) |
| **Cognition** |  |
| MMSE | 28.63 (1.87) |
| MoCA | 27.04 (2.96) |
| Executive function | 0.84 (0.92) |
| Memory | 0.93 (0.97) |
| Language cognition | 0.72 (0.88) |
| Visuo-spatial function | 0.11 (0.75) |
| **Plasma biomarkers** |  |
| GFAP | 2.16 (0.22) |
| NfL | 1.24 (0.17) |
| P-tau217 | -0.79 (0.35) |
| Aβ42/40 | -1.06 (0.05) |

*Notes*: WMH volume was adjusted for total intracranial volume. CSFAβ42, CSF t-tau, CSF p-tau, CSF t-tau/Aβ42 ratio, WMH volume, hippocampal volume, Aβ-PET load, GFAP, NfL, p-tau217, and Aβ42/40 ratio were all log transformed; MMSE and MoCA scores were z transformed; Data were presented as mean (SD), n (%), or median (interquartile range). CSF and plasma biomarkers were measured in pg/mL, brain volume was measured in mL.

Abbreviations: Aβ, amyloid beta; AD, Alzheimer’s disease; APOE, apolipoprotein E; CSF, cerebrospinal fluid; GFAP, glial fibrillary acidic protein; PET, positron emission tomography; MMSE, Mini-Mental State Examination; MoCA, Montreal Cognitive Assessment; NfL, neurofilament light; P-tau, phosphorylated tau; SD, standard deviation; T-tau, total tau; WMH, white matter hyperintensities.

**TABLE S8** Participant lumbar puncture follow-up

|  | N = 86 |
| --- | --- |
| No. of longitudinal lumbar puncture | 1.14 (0.41; 1 to 3) |
| Duration of lumbar puncture follow-up, years | 2.67 (1.17; 2 to 8.5) |

*Notes*: Data were presented as mean (SD; range)

**TABLE S9** Cross-sectional association of plasma biomarkers with imaging markers and cognition in individuals with or without AD.

|  | GFAP | NfL | p-tau217 | Aβ42/40 ratio |
| --- | --- | --- | --- | --- |
|  | β (SE) *p* | β (SE) *p* | β (SE) *p* | β (SE) *p* |
| **Imaging markers** |  |  |  |  |
| **WMH** |  |  |  |  |
| AD− | **0.558 (0.268) 0.039** | **1.473 (0.289) <0.0001** | **0.631 (0.272) 0.022** | 1.049 (1.023) 0.306 |
| AD+ | 0.449 (0.231) 0.054 | 0.154 (0.295) 0.602 | **0.436 (0.144) 0.003** | -0.252 (0.931) 0.787 |
| **Hippocampus** |  |  |  |  |
| AD− | -0.013 (0.026) 0.626 | -0.051 (0.031) 0.098 | -0.022 (0.026) 0.392 | -0.037 (0.099) 0.712 |
| AD+ | -0.038 (0.031) 0.219 | -0.061 (0.037) 0.101 | **-0.067 (0.018) 0.0002** | 0.186 (0.113) 0.103 |
| **Cognition** |  |  |  |  |
| **MMSE** |  |  |  |  |
| AD− | 0.053 (0.256) 0.836 | -0.467 (0.301) 0.122 | -0.155 (0.257) 0.546 | -0.199 (0.970) 0.838 |
| AD+ | **-1.667 (0.517) 0.002** | **-1.528 (0.641) 0.018** | **-1.706 (0.292) <0.0001** | 2.227 (1.982) 0.263 |
| **MoCA** |  |  |  |  |
| AD− | -0152 (0.275) 0.581 | -0.367 (0.319) 0.252 | -0.035 (0.272) 0.898 | -0.021 (1.023) 0.983 |
| AD+ | **-1.475 (0.518) 0.005** | -0.722 (0.648) 0.267 | **-1.781 (0.290) <0.0001** | 0.462 (1.981) 0.816 |
| **Executive function** |  |  |  |  |
| AD− | -0.333 (0.369) 0.368 | -0.795 (0.433) 0.068 | -0.596 (0.368) 0.107 | 0.958 (1.398) 0.494 |
| AD+ | **-1.340 (0.435) 0.002** | -1.063 (0.540) 0.051 | **-1.481 (0.242) <0.0001** | 0.667 (1.661) 0.689 |
| **Memory** |  |  |  |  |
| AD− | -0.182 (0.374) 0.627 | -0.592 (0.441) 0.181 | -0.070 (0.376) 0.853 | 1.012 (1.417) 0.476 |
| AD+ | **-1.277 (0.469) 0.007** | -0.778 (0.584) 0.185 | **-1.905 (0.245) <0.0001** | 1.519 (1.785) 0.396 |
| **Language** |  |  |  |  |
| AD− | -0.363 (0.367) 0.324 | -0.043 (0.436) 0.921 | 0.125 (0.370) 0.735 | 0.092 (1.395) 0.947 |
| AD+ | **-1.222 (0.418) 0.004** | **-1.061 (0.518) 0.004** | **-1.537 (0.227) <0.0001** | 1.248 (1.597) 0.436 |
| **Visuo-spatial function** |  |  |  |  |
| AD− | 0.140 (0.351) 0.690 | -0.323 (0.415) 0.437 | 0.088 (0.352) 0.803 | -0.531 (1.329) 0.690 |
| AD+ | -0.112 (0.388) 0.773 | 0.122 (0.474) 0.797 | -0.410 (0.233) 0.081 | -1.224 (1.442) 0.397 |

Multivariable linear regression models adjusted for age, sex, and APOE *ε*4 status. WMH volume was adjusted for total intracranial volume. WMH volume, hippocampal volume, GFAP, NfL, p-tau217, and Aβ42/40 ratio were all log transformed. MMSE and MoCA scores were z transformed.

Abbreviations: Aβ, amyloid beta; AD, Alzheimer’s disease; GFAP, glial fibrillary acidic protein; MMSE, Mini-Mental State Examination; MoCA, Montreal Cognitive Assessment; NfL, neurofilament light; P-tau, phosphorylated tau; T-tau, total tau; WMH, white matter hyperintensities.

**TABLE S10** Cross-sectional association of plasma biomarkers with imaging markers, CSF t-tau/Aβ42 ratio and cognition in subgroups.

|  | GFAP | NfL | p-tau217 | Aβ42/40 ratio |
| --- | --- | --- | --- | --- |
|  | β (SE) *p* | β (SE) *p* | β (SE) *p* | β (SE) *p* |
| **Imaging markers** |  |  |  |  |
| **WMH** |  |  |  |  |
| AD−WMH− | 0.485 (0.282) 0.087 | **1.364 (0.319) <0.0001** | 0.521 (0.286) 0.070 | 0.284 (1.083) 0.794 |
| AD−WMH+ | -0.107 (0.214) 0.619 | -0.157 (0.239) 0.514 | -0.277 (0.222) 0.220 | 0.298 (0.787) 0.708 |
| AD+WMH− | 0.242 (0.221) 0.275 | -0.374 (0.281) 0.187 | 0.174 (0.133) 0.195 | -0.968 (0.807) 0.234 |
| AD+WMH+ | -0.026 (0.219) 0.907 | 0.109 (0.262) 0.678 | 0.079 (0.155) 0.614 | 0.433 (0.998) 0.666 |
| **Hippocampus** |  |  |  |  |
| AD−WMH− | 0.009 (0.029) 0.766 | -0.037 (0.034) 0.297 | -0.038 (0.028) 0.178 | -0.079 (0.106) 0.457 |
| AD−WMH+ | -0.088 (0.063) 0.174 | -0.092 (0.071) 0.207 | 0.024 (0.070) 0.732 | 0.045 (0.259) 0.862 |
| AD+WMH− | -0.021 (0.041) 0.606 | -0.040 (0.047) 0.397 | **-0.055 (0.022) 0.016** | 0.147 (0.133) 0.272 |
| AD+WMH+ | -0.074 (0.050) 0.147 | -0.103 (0.062) 0.101 | **-0.095 (0.033) 0.005** | 0.258 (0.227) 0.261 |
| **Aβ-PET** |  |  |  |  |
| AD−WMH− | 0.0002 (0.045) 0.997 | -0.027 (0.039) 0.491 | 0.036 (0.041) 0.389 | 0.294 (0.208) 0.171 |
| AD−WMH+ | -0.023 (0.066) 0.738 | -0.100 (0.051) 0.082 | -0.041 (0.054) 0.472 | 0.081 (0.244) 0.746 |
| AD+WMH− | 0.091 (0.050) 0.086 | -0.009 (0.078) 0.908 | **0.101 (0.030) 0.003** | -0.003 (0.194) 0.986 |
| AD+WMH+ | 0.003 (0.060) 0.958 | -0.007 (0.073) 0.922 | **0.101 (0.034) 0.006** | 0.198 (0.283) 0.489 |
| **CSF biomarker** |  |  |  |  |
| **CSF t-tau/Aβ42** |  |  |  |  |
| AD−WMH− | 0.111 (0.059) 0.061 | 0.124 (0.076) 0.106 | **0.217 (0.057) 0.0002** | **-0.911 (0.202) <0.0001** |
| AD−WMH+ | -0.130 (0.096) 0.187 | -0.076 (0.113) 0.511 | -0.023 (0.118) 0.848 | **-0.885 (0.398) 0.036** |
| AD+WMH− | **0.576 (0.121) <0.0001** | **0.357 (0.148) 0.018** | **0.445 (0.052) <0.0001** | -0.070 (0.451) 0.876 |
| AD+WMH+ | **0.324 (0.159) 0.047** | -0.025 (0.204) 0.904 | **0.345 (0.100) 0.001** | -0.876 (0.719) 0.228 |
| **Cognition** |  |  |  |  |
| **MMSE** |  |  |  |  |
| AD−WMH− | -0.071 (0.274) 0.795 | -0.410 (0.335) 0.224 | -0.387 (0.270) 0.155 | 0.476 (1.021) 0.641 |
| AD−WMH+ | 0.117 (0.596) 0.845 | -0.619 (0.663) 0.358 | 0.605 (0.632) 0.346 | -2.450 (2.341) 0.304 |
| AD+WMH− | **-2.410 (0.700) 0.0009** | -1.380 (0.862) 0.114 | **-1.839 (0.373) <0.0001** | 2.054 (2.475) 0.409 |
| AD+WMH+ | -0.199 (0.819) 0.809 | -1.555 (0.994) 0.123 | **-1.286 (0.535) 0.019** | 3.491 (3.653) 0.343 |
| **MoCA** |  |  |  |  |
| AD−WMH− | -0.438 (0.278) 0.118 | -0.530 (0.333) 0.114 | -0.452 (0.268) 0.095 | -0.041 (1.017) 0.968 |
| AD−WMH+ | 0.392 (0.687) 0.573 | 0.168 (0.778) 0.830 | 1.232 (0.645) 0.066 | -0.235 (2.756) 0.932 |
| AD+WMH− | **-1.540 (0.633) 0.017** | -0.723 (0.759) 0.344 | **-1.644 (0.329) <0.0001** | 1.314 (2.171) 0.547 |
| AD+WMH+ | -0.837 (0.929) 0.371 | -0.211 (1.168) 0.857 | **-1.727 (0.594) 0.005** | -1.258 (4.184) 0.765 |
| **Executive function** |  |  |  |  |
| AD−WMH− | -0.634 (0.420) 0.134 | -0.342 (0.520) 0.512 | -0.411 (0.420) 0.330 | -0.923 (1.578) 0.560 |
| AD−WMH+ | 0.493 (0.713) 0.495 | -1.326 (0.773) 0.097 | -1.021 (0.750) 0.184 | **7.759 (2.496) 0.004** |
| AD+WMH− | **-1.623 (0.534) 0.003** | -1.080 (0.644) 0.097 | **-1.492 (0.273) <0.0001** | 0.614 (1.857) 0.742 |
| AD+WMH+ | -0.434 (0.733) 0.556 | -0.543 (0.920) 0.558 | **-1.043 (0.484) 0.035** | 2.051 (3.294) 0.536 |
| **Memory** |  |  |  |  |
| AD−WMH− | -0.612 (0.428) 0.156 | -0.230 (0.531) 0.665 | 0.038 (0.429) 0.930 | 0.433 (1.609) 0.788 |
| AD−WMH+ | 0.629 (0.638) 0.332 | -1.377 (0.686) 0.054 | -0.340 (0.693) 0.628 | 2.839 (2.535) 0.271 |
| AD+WMH− | **-1.466 (0.626) 0.022** | -0.323 (0.755) 0.670 | **-1.743 (0.311) <0.0001** | 1.900 (2.133) 0.376 |
| AD+WMH+ | -0.637 (0.761) 0.406 | -1.408 (0.930) 0.135 | **-2.116 (0.447) <0.0001** | 0.410 (3.441) 0.906 |
| **Language** |  |  |  |  |
| AD−WMH− | -0.472 (0.431) 0.275 | 0.083 (0.532) 0.876 | 0.144 (0.430) 0.739 | -0.408 (1.612) 0.801 |
| AD−WMH+ | -0.243 (0.684) 0.725 | 0.054 (0.772) 0.944 | 0.231 (0.735) 0.756 | 2.326 (2.702) 0.396 |
| AD+WMH− | **-1.447 (0.524) 0.007** | -0.428 (0.638) 0.504 | **-1.428 (0.267) <0.0001** | 0.864 (1.812) 0.635 |
| AD+WMH+ | -0.514 (0.740) 0.490 | **-2.014 (0.883) 0.026** | **-1.601 (0.464) 0.001** | 2.972 (3.319) 0.374 |
| **Visuo-spatial function** |  |  |  |  |
| AD−WMH− | 0.011 (0.393) 0.978 | -0.210 (0.482) 0.665 | 0.310 (0.389) 0.426 | -1.043 (1.460) 0.477 |
| AD−WMH+ | 0.395 (0.793) 0.622 | -0.330 (0.896) 0.715 | -0.451 (0.853) 0.601 | 1.501 (3.169) 0.639 |
| AD+WMH− | -0.042 (0.503) 0.934 | 0.535 (0.583) 0.362 | -0.344 (0.283) 0.229 | -1.544 (1.654) 0.353 |
| AD+WMH+ | -0.154 (0.688) 0.823 | -0.630 (0.848) 0.460 | -0.589 (0.464) 0.209 | -0.168 (3.092)0.957 |

Multivariable linear regression models adjusted for age, sex, and APOE *ε*4 status. WMH volume was adjusted for total intracranial volume. WMH volume, hippocampal volume, CSF t-tau/Aβ42 ratio, Aβ-PET load, GFAP, NfL, p-tau217, and Aβ42/40 ratio were all log transformed. MMSE and MoCA scores were z transformed.

Abbreviations: Aβ, amyloid beta; AD, Alzheimer’s disease; CSF, cerebrospinal fluid; GFAP, glial fibrillary acidic protein; PET, positron emission tomography; MMSE, Mini-Mental State Examination; MoCA, Montreal Cognitive Assessment; NfL, neurofilament light; P-tau, phosphorylated tau; T-tau, total tau; WMH, white matter hyperintensities.

**TABLE S11** Longitudinal associations of baseline plasma biomarker-time interaction with imaging markers and cognition in in individuals with or without AD.

|  | GFAP*time | NfL*time | p-tau217*time | Aβ42/40 ratio*time |
| --- | --- | --- | --- | --- |
|  | β (SE) *p* | β (SE) *p* | β (SE) *p* | β (SE) *p* |
| **Imaging markers** |  |  |  |  |
| **WMH** |  |  |  |  |
| AD− | 0.034 (0.046) 0.465 | -0.063 (0.060) 0.295 | 0.047 (0.058) 0.417 | 0.088 (0.207) 0.671 |
| AD+ | **0.071 (0.028) 0.011** | **0.127 (0.037) 0.0008** | **0.047 (0.016) 0.004** | -0.241 (0.132) 0.070 |
| **Hippocampus** |  |  |  |  |
| AD− | 0.002 (0.004) 0.661 | -0.008 (0.005) 0.118 | 0.002 (0.005) 0.748 | -0.192 (0.018) 0.294 |
| AD+ | **-0.014 (0.004) 0.002** | **-0.014 (0.006) 0.020** | **-0.008 (0.003) 0.001** | **0.042 (0.021) 0.045** |
| **Cognition** |  |  |  |  |
| **MMSE** |  |  |  |  |
| AD− | 0.054 (0.069) 0.432 | 0.038 (0.092) 0.681 | -0.103 (0.078) 0.186 | -0.051 (0.280) 0.855 |
| AD+ | -0.054 (0.128) 0.672 | 0.077 (0.139) 0.580 | **-0.300 (0.076) <0.0001** | **0.948 (0.437) 0.031** |
| **MoCA** |  |  |  |  |
| AD− | 0.137 (0.111) 0.218 | -0.002 (0.141) 0.986 | 0.063 (0.121) 0.603 | -0.857 (0.442) 0.054 |
| AD+ | -0.171 (0.137) 0.212 | -0.036 (0.150) 0.810 | **-0.394 (0.083) <0.0001** | **1.464 (0.486) 0.003** |
| **Executive function** |  |  |  |  |
| AD− | -0.038 (0.080) 0.636 | -0.003 (0.105) 0.979 | 0.020 (0.090) 0.824 | -0.182 (0.320) 0.570 |
| AD+ | -0.086 (0.056) 0.127 | **-0.121 (0.061) 0.049** | **-0.118 (0.034) 0.0005** | 0.793 (0.192) **<0.0001** |
| **Memory** |  |  |  |  |
| AD− | -0.083 (0.066) 0.208 | -0.051 (0.087) 0.556 | -0.089 (0.074) 0.232 | **-0.578 (0.261) 0.028** |
| AD+ | 0.014 (0.057) 0.803 | **0.127 (0.061) 0.038** | **-0.140 (0.034) <0.0001** | **0.593 (0.193) 0.002** |
| **Language** |  |  |  |  |
| AD− | 0.046 (0.073) 0.530 | -0.079 (0.097) 0.413 | -0.205 (0.082) 0.012 | -0.169 (0.296) 0.567 |
| AD+ | -0.108 (0.063) 0.089 | **-0.091 (0.070) 0.190** | **-0.162 (0.038) <0.0001** | 0.947 (0.217) **<0.0001** |
| **Visuo-spatial function** |  |  |  |  |
| AD− | -0.0008 (0.096) 0.993 | -0.018 (0.127) 0.888 | -0.042 (0.108) 0.695 | 0.388 (0.010) 0.992 |
| AD+ | **-0.207 (0.077) 0.007** | -0.132 (0.084) 0.116 | **-0.136 (0.047) 0.004** | 0.433 (0.270) 0.109 |

*Notes*: WMH volume was adjusted for total intracranial volume. WMH volume, hippocampal volume, GFAP, NfL, p-tau217, and Aβ42/40 ratio were all log transformed; MMSE and MoCA scores were z transformed. Liner mixed models adjusted age, sex, APOE *ε*4 carrier status, baseline cognitive score, and interaction of all predictors with time.

Abbreviations: Aβ, amyloid beta; AD, Alzheimer’s disease; GFAP, glial fibrillary acidic protein; MMSE, Mini-Mental State Examination; MoCA, Montreal Cognitive Assessment; NfL, neurofilament light; P-tau, phosphorylated tau; WMH, white matter hyperintensities.

**TABLE S12** Longitudinal associations of baseline plasma biomarker-time interaction with cognition in subgroups.

|  | GFAP*time | NfL*time | p-tau217*time | Aβ42/40*time |
| --- | --- | --- | --- | --- |
|  | β (SE) *p* | β (SE) *p* | β (SE) *p* | β (SE) *p* |
| **MMSE** |  |  |  |  |
| AD−WMH− | 0.046 (0.086) 0.592 | 0.076 (0.110) 0.491 | -0.086 (0.085) 0.314 | -0.095 (0.324) 0.770 |
| AD−WMH+ | 0.163 (0.165) 0.326 | 0.052 (0.211) 0.805 | -0.183 (0.248) 0.463 | 0.220 (0.643) 0.733 |
| AD+WMH− | -0.071 (0.143) 0.622 | 0.139 (0.154) 0.367 | **-0.288 (0.089) 0.001** | 0.803 (0.493) 0.105 |
| AD+WMH+ | 0.189 (0.330) 0.567 | 0.007 (0.383) 0.986 | -0.215 (0.179) 0.234 | -0.833 (1.160) 0.474 |
| **MoCA** |  |  |  |  |
| AD−WMH− | 0.157 (0.128) 0.221 | 0.171 (0.164) 0.298 | 0.176 (0.130) 0.176 | -0.483 (0.488) 0.323 |
| AD−WMH+ | 0.053 (0.285) 0.853 | -0.394 (0.340) 0.249 | -0.328 (0.394) 0.408 | -1.716 (1.088) 0.119 |
| AD+WMH− | -0.135 (0.140) 0.338 | 0.100 (0.152) 0.513 | **-0.292 (0.088) 0.001** | **1.082 (0.519) 0.039** |
| AD+WMH+ | -0.177 (0.411) 0.667 | **-1.653 (0.544) 0.003** | **-0.845 (0.222) 0.0002** | 2.309 (1.502) 0.126 |
| **Executive function** |  |  |  |  |
| AD−WMH− | 0.053 (0.102) 0.602 | 0.132 (0.131) 0.312 | 0.052 (0.102) 0.610 | -0.327 (0.385) 0.396 |
| AD−WMH+ | **-0.374 (0.169) 0.030** | -0.438 (0.223) 0.054 | -0.147 (0.262) 0.577 | 0.301 (0.675) 0.657 |
| AD+WMH− | -0.093 (0.069) 0.182 | -0.114 (0.075) 0.129 | **-0.142 (0.044) 0.001** | **0.783 (0.236) 0.001** |
| AD+WMH+ | 0.014 (0.131) 0.915 | -0.139 (0.152) 0.361 | -0.070 (0.071) 0.326 | 0.719 (0.459) 0.120 |
| **Memory** |  |  |  |  |
| AD−WMH− | -0.008 (0.083) 0.919 | 0.053(0.105) 0.616 | -0.038 (0.082) 0.644 | -0.524 (0.308) 0.091 |
| AD−WMH+ | -0.188 (0.149) 0.212 | -0.209 (0.193) 0.283 | -0.121 (0.224) 0.591 | -0.574 (0.573) 0.320 |
| AD+WMH− | 0.031 (0.067) 0.642 | 0.191 (0.071) 0.007 | **-0.100 (0.042) 0.018** | 0.362 (0.230) 0.118 |
| AD+WMH+ | 0.045 (0.135) 0.737 | -0.009 (0.157) 0.956 | **-0.189 (0.073) 0.010** | 0.816 (0.472) 0.086 |
| **Language** |  |  |  |  |
| AD−WMH− | 0.051 (0.097) 0.597 | -0.062 (0.124) 0.620 | **-0.213 (0.096) 0.027** | 0.103 (0.366) 0.780 |
| AD−WMH+ | 0.049 (0.141) 0.731 | -0.177 (0.180) 0.328 | -0.333 (0.209) 0.115 | **-1.192 (0.513) 0.023** |
| AD+WMH− | -0.144 (0.077) 0.061 | -0.077 (0.084) 0.357 | **-0.196 (0.049) <0.0001** | **1.039 (0.265) 0.0001** |
| AD+WMH+ | 0.167 (0.144) 0.249 | -0.083 (0.167) 0.619 | -0.081 (0.079) 0.303 | 0.258 (0.511) 0.615 |
| **Visuo-spatial function** |  |  |  |  |
| AD−WMH− | -0.060 (0.112) 0.594 | 0.045 (0.143) 0.752 | 0.041 (0.112) 0.716 | 0.015 (0.424) 0.973 |
| AD−WMH+ | 0.071 (0.265) 0.790 | -0.176 (0.332) 0.598 | -0.686 (0.389) 0.081 | 0.258 (1.026) 0.802 |
| AD+WMH− | **-0.185 (0.090) 0.042** | -0.104 (0.098) 0.294 | **-0.134 (0.058) 0.022** | 0.314 (0.324) 0.333 |
| AD+WMH+ | 0.840 (0.189) 0.657 | -0.199 (0.218) 0.361 | -0.019 (0.104) 0.854 | 1.182 (0.663) 0.076 |

*Notes*: WMH volume was adjusted for total intracranial volume. WMH volume, hippocampal volume, GFAP, NfL, p-tau217, and Aβ42/40 ratio were all log transformed; MMSE and MoCA scores were z transformed. Liner mixed models adjusted age, sex, APOE *ε*4 carrier status, baseline cognitive score, and interaction of all predictors with time. Abbreviations: Aβ, amyloid beta; AD, Alzheimer’s disease; GFAP, glial fibrillary acidic protein; MMSE, Mini-Mental State Examination; MoCA, Montreal Cognitive Assessment; NfL, neurofilament light; P-tau, phosphorylated tau; WMH, white matter hyperintensities.

**Table S13** Discriminative performance of baseline plasma biomarkers.

| **Predictor** | **AUC** | **AUC 95% CI** | **Specificity** | **Sensitivity** | **Accuracy** | **NPV** | **PPV** | **Optimal cut point** |
| --- | --- | --- | --- | --- | --- | --- | --- | --- |
| **AD−WMH− vs AD+WMH+** | | | | | | | | |
| GFAP | 0.895 | 0.848-0.942 | 0.87 | 0.809 | 0.83 | 0.809 | 0.87 | **153.7** |
| NfL | 0.848 | 0.795-0.901 | 0.739 | 0.824 | 0.795 | 0.824 | 0.739 | **20.1** |
| p-tau217 | 0.985 | 0.971-0.999 | 0.928 | 0.969 | 0.955 | 0.969 | 0.928 | **0.199** |
| Aβ42/40 | 0.894 | 0.852-0.957 | 0.957 | 0.718 | 0.2 | 0.282 | 0.043 | **0.089** |

AUC, specificity, sensitivity, accuracy, NPV, and PPV of plasma biomarkers (Youden’s index-optimized cutoffs) for distinguishing AD+WMH+ from AD-WMH-.

Abbreviations: Aβ, amyloid beta; AD, Alzheimer’s disease; AUC: area under the curve; CSF, cerebrospinal fluid; GFAP, glial fibrillary acidic protein; NfL, neurofilament light; NPV, negative predictive value; PPV, positive predictive value; P-tau, phosphorylated tau; WMH, white matter hyperintensities.

**TABLE S14** Hazard Ratios (95%CI) for CSVD in normal control.

|  | Continuous Variable | |  | | Categorical Variable* | |
| --- | --- | --- | --- | --- | --- | --- |
|  | HR (95%CI) | *p* |  | HR (95%CI) | | *p* |
| **Univariable model** |  |  |  |  | |  |
| GFAP, per unit | **1.017 (1.002-1.034)** | **0.030** | GFAP**†** | 3.335 (0.763-14.574) | | 0.066 |
| NfL, per unit | 0.987 (0.910-1.070) | 0.745 | NfL**†** | 0.530 (0.149-1.890) | | 0.317 |
| p-tau217, per 0.1 unit | 1.230 (0.250-6.057) | 0.799 | p-tau217 | 4.054 (0.839-19.577) | | 0.081 |
| Aβ42/40, per 0.01 unit | 0.706 (0.336-1.483) | 0.358 | Aβ42/40 | 2.836 (0.604-13.321) | | 0.187 |
| **Multivariable model** |  |  |  |  | |  |
| GFAP, per unit | **1.018 (1.001-1.036)** | **0.042** | GFAP**†** | 3.019 (0.647-0.930) | | 0.113 |
| NfL, per unit | 0.896 (0.781-1.029) | 0.121 | NfL**†** | 0.206 (1.037-2.884)‡ | | 0.058 |
| p-tau217, per 0.1 unit | 0.682 (0.093-5.016) | 0.707 | p-tau217 | 3.513 (0.625-19.763) | | 0.154 |
| Aβ42/40, per 0.01 unit | 0.703 (0.358-1.378) | 0.304 | Aβ42/40 | 3.025 (0.635-14.402) | | 0.164 |

*Notes*: Multivariable models adjusted age and sex. Plasma biomarkers were measured in pg/mL. Biomarker cutpoints were identified using the survminer package in R.

Abbreviations: Aβ, amyloid beta; CI, Confidence interval; GFAP, glial fibrillary acidic protein; HR, Hazard ratio; NfL, neurofilament light; P-tau, phosphorylated tau; T-tau, total tau.

* Cox regression models were fitted using Firth's bias-reduced penalized likelihood method.

**†**Profile likelihood CI.
