# Supplementary material for: Associations of plasma biomarkers with longitudinal co-pathologies in Alzheimer’s disease and cerebral small vessel disease comorbidity
Source: J Prev Alzheimers Dis. 2026 Jan 1;13(2):100449. doi: 10.1016/j.tjpad.2025.100449 (PMC12869035; doi:10.1016/j.tjpad.2025.100449)
Supplement: Supplementary file 2 [file mmc2.docx]

**Figure S1**


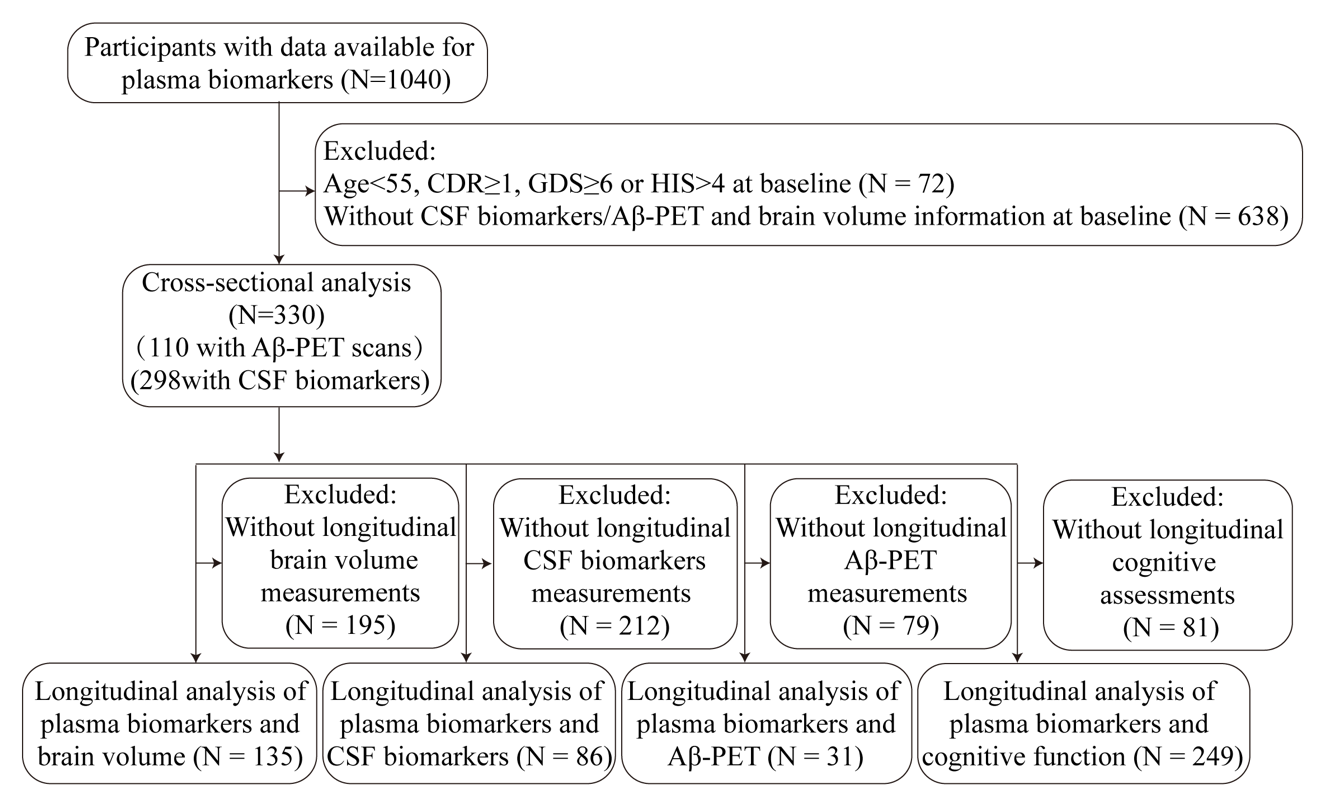
**Figure S1** Flowchart of participant data selection process. Aβ, amyloid beta; APOE, apolipoprotein E; CDR, clinical dementia rating; CSF, cerebrospinal fluid; PET, positron emission tomography; HIS, Hachinski ischemic score; GDS, geriatric depression scale.

**
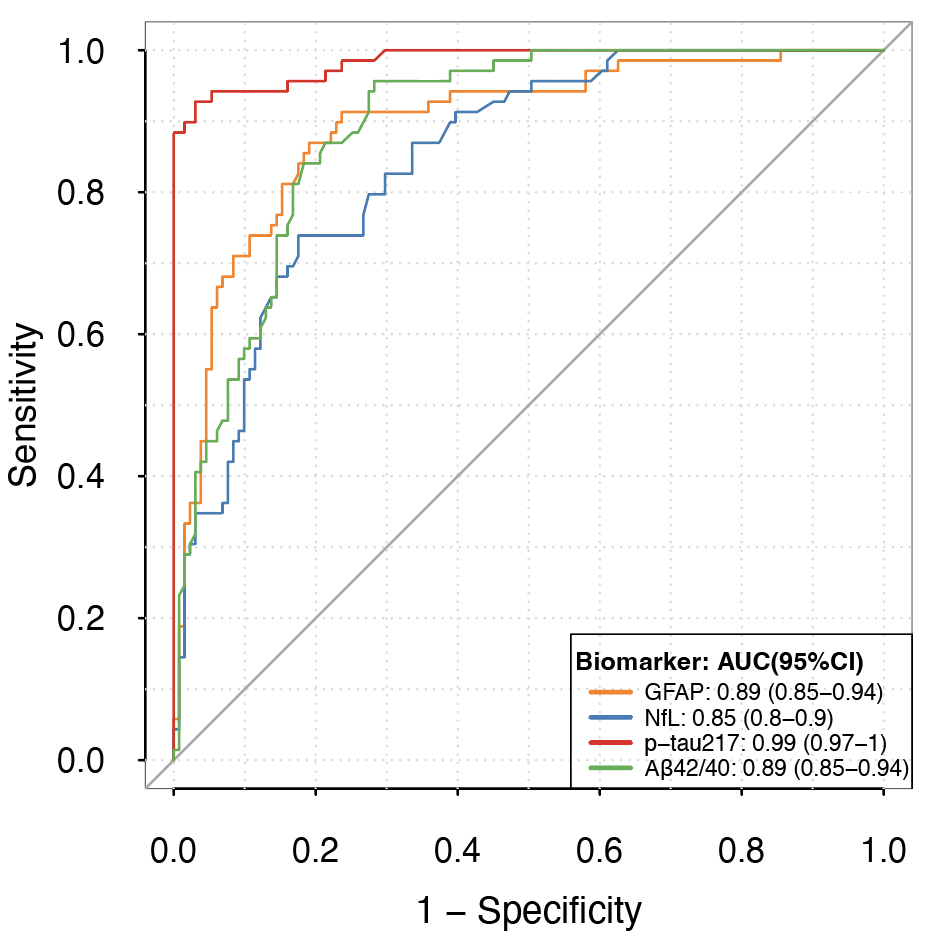
Figure S2**

**Figure S2** Receiver operating characteristic (ROC) curves for distinguishing between AD−WMH− vs AD+WMH+. ROC curves of plasma GFAP, NfL, p-tau217, Aβ42/40 ratio, and combined plasma biomarkers. Aβ, amyloid beta; AUC, area under the curve; CI, confidence interval. GFAP, glial fibrillary acidic protein; NfL, neurofilament light; P-tau, phosphorylated tau.

**Figure S3**

**
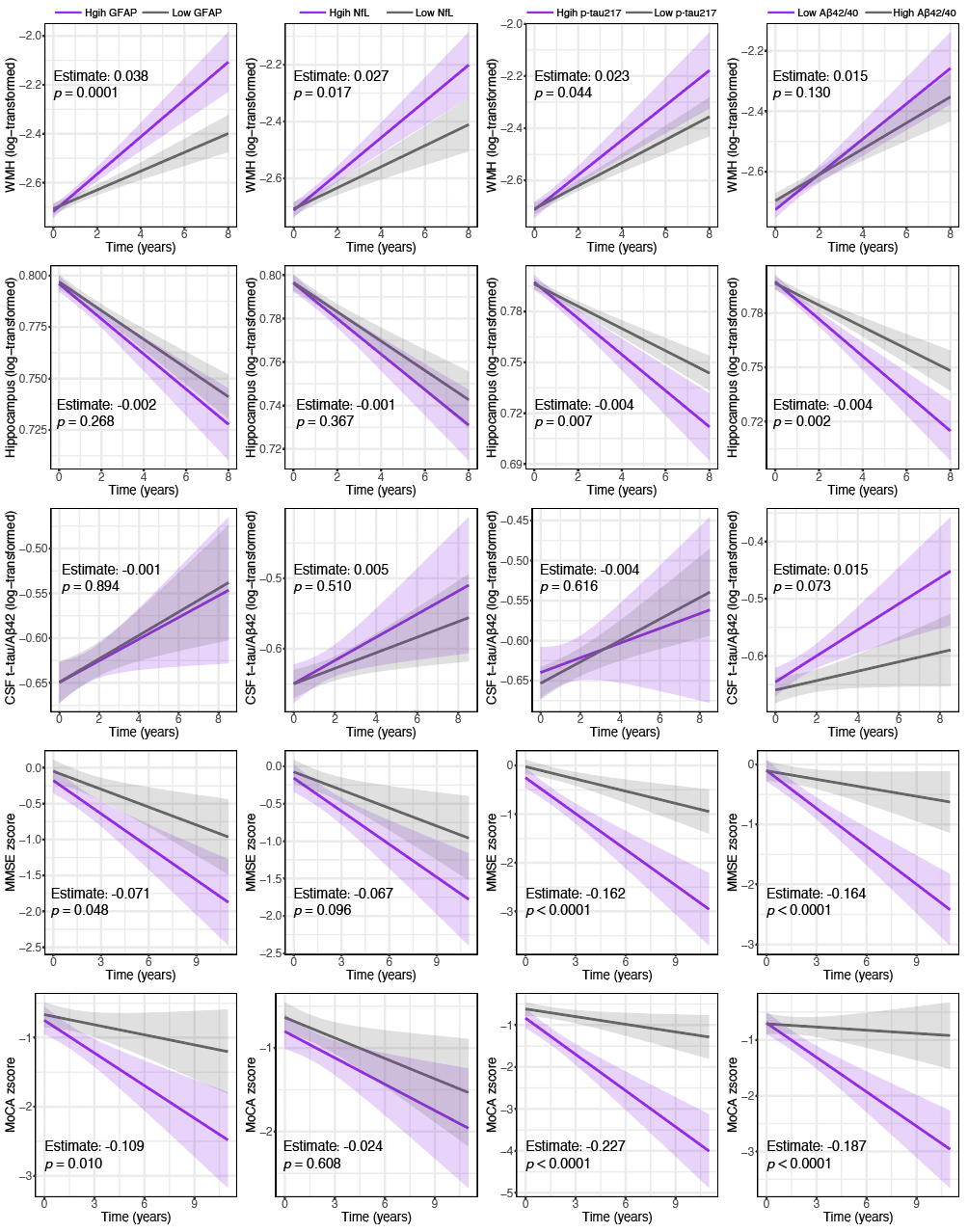
**(continued)


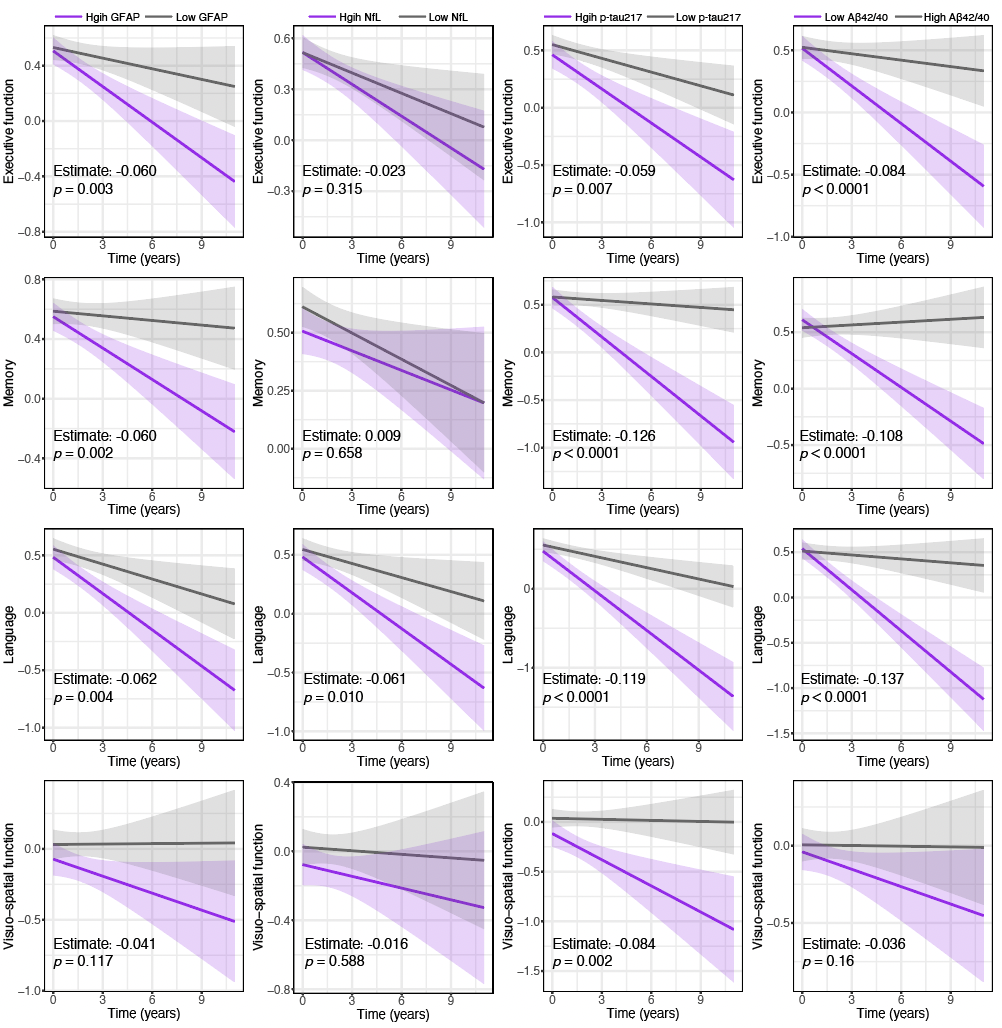


**Figure S3** Longitudinal associations between baseline plasma biomarker (high vs low) with brain structural changes, CSF Aβ42/40 ratio, and cognition .

*Note*: High vs low plasma biomarkers based on optimal cut points achieved from Youden’s index (AD-WMH- vs AD+WMH+).

Abbreviations: Aβ, amyloid beta; CSF, cerebrospinal fluid; GFAP, glial fibrillary acidic protein; MMSE, Mini-Mental State Examination; MoCA, Montreal Cognitive Assessment; NfL, neurofilament light; P-tau, phosphorylated tau; T-tau, total tau; WMH, white matter hyperintensities.

**Figure S4**


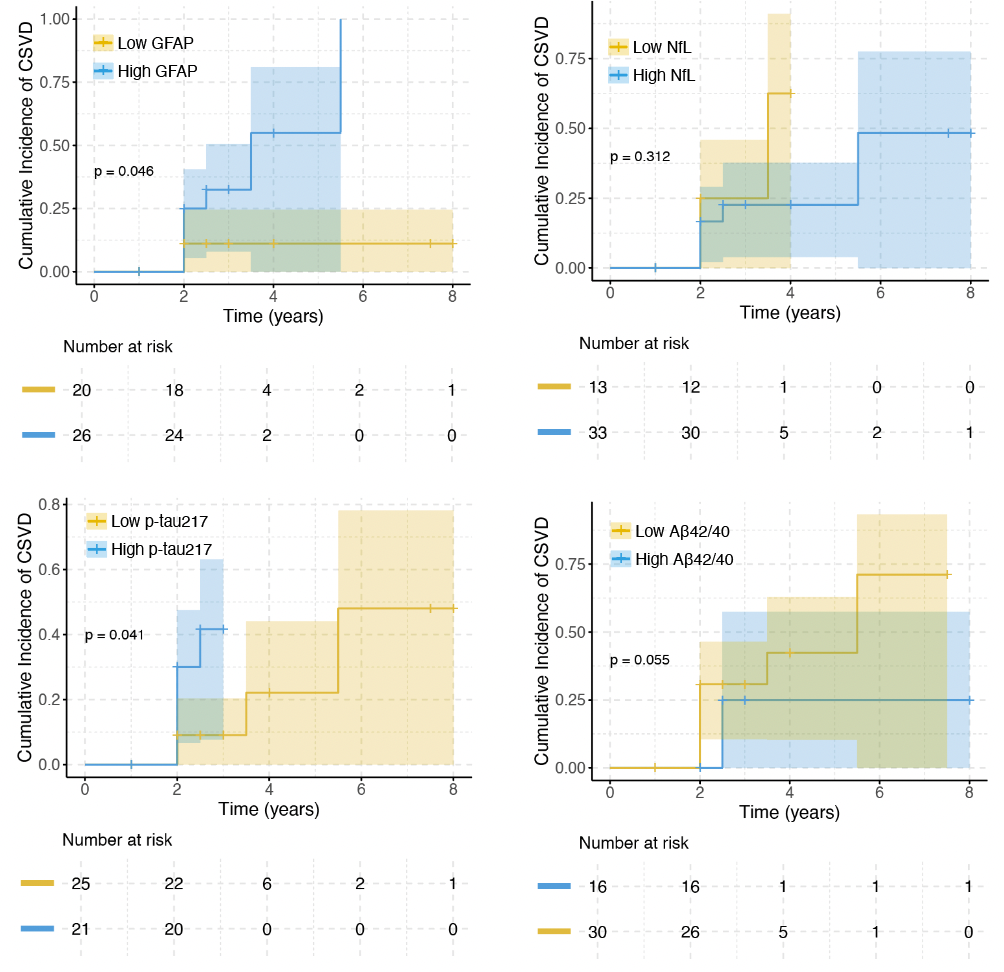


**Figure S4** Kaplan–Meier curve of CSVD incidence in normal control. Biomarker cutpoints were identified using the survminer package in R. Group differences were evaluated via log-rank tests. Aβ, amyloid beta; GFAP, glial fibrillary acidic protein; NfL, neurofilament light; P-tau, phosphorylated tau.
